# Supplementary material for: Development of X-SIAGA: A disease X and outbreak preparedness intervention for indigenous households in Selangor, Malaysia
Source: PLoS One. 2026 Mar 30;21(3):e0345785. doi: 10.1371/journal.pone.0345785 (PMC13035154; doi:10.1371/journal.pone.0345785)
Supplement: S1 Table — Search strategy and terms used for literature review for needs assessment. (PDF) [file pone.0345785.s001.pdf]

S1 Table. Search strategy and terms

Search strategy and terms used for the literature review.

Search Set		Search Terms (Title)
Population (P)	#1	indigenous people, aboriginals, orang asli, household, households, family, families, individual, individuals, personal, community, communities, public
Issue/Exposure (I)	#2	outbreak, outbreaks, epidemic, epidemics, infectious disease, infectious diseases, communicable disease, communicable diseases, Disease X, emerging disease, emerging diseases, disaster, disasters, health emergency, health emergencies
Outcome (O)	#3	preparedness, readiness
Final Search		((#1) AND (#2)) AND (#3)
