# Supplementary material for: Development of X-SIAGA: A disease X and outbreak preparedness intervention for indigenous households in Selangor, Malaysia
Source: PLoS One. 2026 Mar 30;21(3):e0345785. doi: 10.1371/journal.pone.0345785 (PMC13035154; doi:10.1371/journal.pone.0345785)
Supplement: S2 File — Summary of literature review and secondary data analysis findings for needs assessment. (PDF) [file pone.0345785.s002.pdf]

S2 File. Literature review and secondary data analysis results.

S2.1 Table. Evidence table for the literature review on preparedness and vulnerabilities to outbreaks among the Orang Asli.

	**Author (Year)**	**Type of Disaster**	**Study Design**	**Key Findings and Interpretation**
1.	Dorasamy et al. (2010)	General disaster	Cross-sectional	1. Majority aged 31-40, educated with income RM1000-RM4999.<br>2. Main information sources: television, internet, newspapers; moderate preparedness perception.<br>3. Identified need: Increased disaster preparedness education/training.<br>4. Causes: Lack of access to info/training, overreliance on media, insufficient knowledge, limited policy emphasis, inadequate resources, low public engagement.<br>5. Immediate effects: Loss of life, property damage, disrupted life/economic activities, increased demand for emergency services, psychological trauma.<br>6. Impact: Reduced economic growth, increased healthcare costs, reduced quality of life, heightened vulnerability, potential lack of emergency preparedness among Malaysians.
2.	Bisan & Goh (2015)	General disaster	Scoping review	1. Hidden inequalities exacerbate health service access gaps during disasters.<br>2. Failure in disaster planning and implementation causes service deficiencies.<br>3. Structural inequalities perpetuate social/environmental injustices.<br>4. Immediate effect: Indigenous communities face food insecurity, altered crop patterns, and health quality impacts.<br>5. Impact: Environmental degradation and climate effects worsen health, increase vulnerability, and deepen inequalities among indigenous communities.
3.	Sadeka, Mohamad, & Sarkar	Flood disaster	Qualitative study	1. Social capital is crucial for disaster preparedness among Orang Asli people in Tasik

	(2020)			Chini, Malaysia.<br>2. Orang Asli rely on strong community bonds for support during disasters but lack access to early warnings and information.<br>3. Socio-economic challenges hinder Orang Asli's disaster preparedness despite understanding hazards and risks.<br>4. Direct causes: Lack of preparedness, limited access to resources/information, and exclusion from mainstream disaster efforts.<br>5. Root causes: Historical socio-political marginalization, lack of recognition of indigenous knowledge, geographic vulnerability.<br>6. Immediate effects: Loss of lives, infrastructure damage, service disruptions, community displacement.<br>7. Impact: Economic losses, social/psychological impacts, increased vulnerability, marginalized communities, widened inequalities, undermined trust and social cohesion, hindered sustainable development and SDG progress.
4.	Sadeka, Mohamad, Sarkar, et al. (2020)	Flood disaster	Qualitative study	1. Orang Asli, a low-income community in Malaysia, faces significant flood-related challenges.<br>2. Despite awareness of risks, limited interest in disaster risk reduction programs exists.<br>3. Community lacks preparedness but holds strong familial and neighborly ties.<br>4. Desire to protect culture could aid in building a resilient community.<br>5. Active participation in workshops can improve disaster knowledge.<br>6. Direct causes: Limited resources, participation, awareness, socio-economic status, infrastructure, and government support.<br>7. Root causes: Marginalization, discrimination, limited political power, and geographic vulnerability.<br>8. Immediate effects: Loss of life, property damage, service disruptions, displacement, livelihood loss.

				9. Impact: Psychological trauma, economic losses, social dislocation, environmental degradation, increased vulnerability.<br>10. Continued vulnerability of Orang Asli to disasters, risk of life/property loss, economic losses, social dislocation, environmental degradation, increased future vulnerability, continued marginalization.
5.	Shafii et al. (2023)	Outbreak	Cross-sectional	1. Floods escalate bacterial populations, heightening waterborne disease exposure.<br>2. Midstream flood victims in urbanized areas face heightened infection vulnerability.<br>3. Direct causes: Flooding during monsoon seasons raises water levels and bacterial populations, increasing waterborne disease risks. Urbanization in midstream areas intensifies vulnerability.<br>4. Root causes: Ineffective flood management, insufficient climate change adaptation causing increased flood risks.<br>5. Immediate effects: Elevated waterborne disease risk during monsoons, vulnerability in urbanized midstream areas, potential disease outbreaks.<br>6. Impact: Limitations in flood risk model may impede risk management. Inadequate integration in flood warning systems could hinder early action. Insufficient study of floodwater microorganisms limits understanding of health impact.
6.	Nungsari et al. (2021)	Outbreak	Qualitative study	1. Vulnerable populations face challenges: limited healthcare, education, basic needs.<br>2. Tailored, culturally sensitive aid approaches for diverse vulnerable groups are crucial.<br>3. Direct cause: Lack of tailored, culturally sensitive aid approaches.<br>4. Root causes: Pre-existing inequalities, discriminatory policies/practices, discriminatory welfare policies.<br>5. Immediate effect: Limited access to healthcare, education, basic needs.<br>6. Impact: Lack of trust, affects health/well-being of individuals and communities.
7.	Rosnon et al. (2022)	Outbreak	Cross-sectional	1. Orang Asli elderly in Peninsular Malaysia showed moderate Covid-19 knowledge (mean score: 7.5/13).

				2. Positive attitudes towards Covid-19 prevention, yet some misconceptions/negative attitudes on vaccination.<br>3. Good preventive practices, but gaps in social distancing and avoiding crowded places.<br>4. Demographic factors (age, gender, education, income) influenced KAP, requiring targeted interventions.<br>5. Some respondents willing to compromise indigenous dietary practices due to Covid-19 concerns.<br>6. Direct causes: Limited Covid-19 knowledge, negative vaccination attitudes, challenges in home isolation, limited access to accurate information.<br>7. Root causes: Lack of detailed outbreak information, government trust issues.<br>8. Immediate effects: Higher infection risk, hindered vaccination efforts.<br>9. Impact: Long-term health outcomes, affecting vaccination rates/herd immunity, contributing to disease spread.
8.	Barbier (2021)	Outbreak	Modelling	1. Smaller wildlife habitats increase human-wildlife contact, heightening disease outbreak risks.<br>2. Land conversion and habitat fragmentation raise zoonotic disease transmission risk.<br>3. Urgent need for research, quantification, and policy implications to avert major zoonotic pandemics.<br>4. Direct causes: Habitat loss, fragmentation due to deforestation, urbanization, agricultural expansion, increased human-wildlife contact.<br>5. Root causes: Unsustainable economic growth prioritizing short-term gains, failure to recognize human-ecological interdependence, neglecting biodiversity's role in human health.<br>6. Immediate effects: Higher zoonotic disease outbreak risk, potential for severe pandemics like COVID-19.<br>7. Impact: Biodiversity loss, heightened disease outbreak risks, more frequent/severe

				pandemics, economic and social costs, long-term consequences for human well-being and the environment.
9.	Chinnasamy et al. (2021)	Outbreak	Narrative review	1. Indigenous communities in Malaysia face unique pandemic challenges: limited healthcare access, resource scarcity, cultural barriers.<br>2. Effective crisis communication and relief management, culturally sensitive and participatory, are crucial for addressing indigenous communities' needs during the pandemic. Collaboration between government, NGOs, and community leaders is essential.<br>3. Direct causes: Inadequate crisis strategies, limited pre-pandemic healthcare/resources, lack of pandemic information/resources, cultural and linguistic barriers to communication.<br>4. Root causes: Systemic inequalities, historical marginalization, lack of political will to address indigenous needs in public health policies, power struggles, strong cultural bonds within indigenous communities.<br>5. Immediate effects: Confusion, fear, mistrust of healthcare providers, non-compliance with public health guidelines, ongoing virus transmission.<br>6. Impact: Heightened virus spread, increased morbidity/mortality among indigenous peoples, widening social/economic disparities with the general population.
10.	Idrus et al. (2021)	Outbreak	Situation report	1. Orang Asli vulnerability to pandemic due to lost territories and forest destruction.<br>2. Wellness rituals/prayers performed for community health and virus prevention.<br>3. Direct causes: COVID-19 outbreak necessitating protective measures, compounded by land displacement/environmental destruction.<br>4. Root causes: Lack of respect for Orang Asli's traditional knowledge/territory control, leading to vulnerability and disrupted indigenous practices.<br>5. Immediate effects: Loss of work/agricultural sales, effective use of traditional knowledge for COVID-19 mitigation, vulnerability from lost territories/destruction.<br>6. Impact: Restricted income sources, compromised epidemic response due to weakened territorial control, potential loss of traditions/knowledge, reduced resilience for future

				health crises, loss of potential solutions to global health issues.
11.	Morand & Lajaunie (2021)	Outbreak	Modelling	1. Disease outbreaks prevail in tropical regions due to high forest cover and deforestation rates.<br>2. Direct causes: Forest conversion for agriculture, logging, infrastructure development, and increasing land demands.<br>3. Root causes: Prioritizing short-term gains over sustainability, inequitable resource allocation, weak governance, and policy failures.<br>4. Immediate effects: Habitat loss, soil erosion, increased greenhouse gas emissions, disrupted water cycles.<br>5. Impact: Reduced biodiversity, amplified climate change risks, diminished resource availability, heightened disease transmission risks, and public health threats.
12.	Mohd Rosman et al. (2020)	Outbreak	Qualitative study	1. Orang Asli community faces health disparities compared to non-aboriginal groups.<br>2. Factors contributing to poor health include inadequate education, ancestral superstitions, and limited access to health information.<br>3. Issues are interconnected, with improvements in one area benefiting others.<br>4. Direct causes: Low immunization coverage for the Batek tribe, especially for measles vaccinations; Nomadic lifestyle of the Batek tribe challenges healthcare provision; Deforestation impacting water sources in Kampung Kuala Koh.<br>5. Root causes: Government's ignorance of illegal mining activities; Inadequate education, superstitions, and limited health facilities; Failure to recognize and support indigenous practices.<br>6. Immediate effects: Food shortage due to environmental impact on hunting and gathering areas; Potential secondary outbreaks in other Orang Asli groups from affected individuals.<br>7. Impact: Malnutrition and worsening health in the indigenous population; Deterioration in overall health outcomes for the Orang Asli community.
13.	Fahmi et al. (2020)	Outbreak	Narrative review	1. Majority in Kampung Peta reliant on tourism, affected by Covid-19.

				2. Direct Causes: Restricted Movement Order enforced due to COVID-19 outbreak.<br>3. Root Causes: Local dependency on limited resources.<br>4. Immediate Effects: Substantial tourism decline and financial strain.<br>5. Impacts: Prolonged COVID-19 impact, sustained income loss, financial strain, potential food insecurity, and policy inattention worsening issues.
14.	Hawkes et al. (2019)	Outbreak	Cross-sectional	1. Anopheles mosquitoes varied across habitats; forest edges had the most diverse species.<br>2. Human intrusion into forests may heighten zoonotic malaria transmission by altering mosquito populations.<br>3. Causes: Deforestation, land changes altering vector compositions, creating new habitats, affecting microclimate and host availability.<br>4. Root Causes: Unsustainable land practices causing habitat loss, impacting vector populations, combined with ineffective malaria control measures and poverty.<br>5. Immediate Effects: Increased zoonotic malaria risk due to human intrusion altering mosquito compositions, affecting malaria control.<br>6. Impact: Biodiversity impacted by deforestation altering mosquito populations, potentially increasing morbidity, mortality, and economic burdens in malaria-endemic regions.
15.	Liew et al. (2018)	Outbreak	Cross-sectional	1. 216 malaria cases detected; Plasmodium vivax causing majority (83.8%).<br>2. Outbreak affected all five villages; highest attack rate in Kampung Senangit (13.5%), outbreak peaked in January 2017, controlled by April 2017.<br>3. Cross-sectional survey found 13.7% malaria prevalence, predominantly P. vivax.<br>4. Causes: Forest activities, lack of insecticide treated net use, poor housing, limited healthcare access, inadequate resources, low awareness of prevention, ineffective control measures, changes in local ecology.<br>5. Root Causes: Poverty, inadequate housing, deforestation, population mobility.<br>6. Immediate Effect: Morbidity and mortality associated with the disease.

				7. Impact: Risk of larger-scale epidemic spread, potential endemicity, perpetuating poverty-disease cycle.
16.	Mohd Radi et al. (2018)	Outbreak	Cross-sectional	1. Vulnerability analysis highlighted poor sanitation, high-risk activities as susceptibility factors.<br>2. Direct Causes: Exposure to contaminated environments, increased human-animal-leptospira interactions, displacement during flooding leading to overcrowding and poor sanitation.<br>3. Root Causes: Climate change inducing frequent flooding in Malaysia, elevating leptospirosis outbreak risk.<br>4. Immediate Effect: Increased disease cases, potentially severe health outcomes like multiorgan failure and death.<br>5. Impact: Cyclical outbreaks post-floods, rising leptospirosis incidence, healthcare system strain due to repeated severe outbreaks.
17.	Venugopalan et al. (2004)	Outbreak	Investigation report	1. Hepatitis A outbreak occurred between April and October 2002, affecting 51 cases in 2 Orang Asli and 2 Malay villages in Hulu Langat.<br>2. Causes: River contamination from human sewage, poor sanitation, open defecation, inadequate refuse disposal, and limited drinking water and latrine coverage.<br>3. Root Causes: Lack of basic life amenities - drinking water, sanitary toilets, refuse disposal, coupled with traditional beliefs against toilet usage.<br>4. Immediate Effect: Health complications arising from Hepatitis A infection.<br>5. Impact: Continued disease spread within and beyond affected areas, increased morbidity, mortality, healthcare costs.

S2.2 Table. Evidence table for the literature review on community-based programs, simulation exercise and game-based learning for outbreak and disaster preparedness.

	Author (Year)	Type of Hazard	Intervention Level	Tool	Key Findings and Interpretation
18.	Ochta Pebriyanti et al. (2023)	General hazard	Individual	Systematic review	1. Communities need to be empowered in the role of disaster management in rural areas.<br>2. Community-based risk management can: (1) reduce disaster risk, (2) increase community resilience to disasters, (3) enhance community capacity for self-preparation, (4) increase community knowledge about disasters.<br>3. Optimizing the role of the community in disaster management is crucial to minimize potential disaster risks.<br>4. There is a significant need for inclusive and community-specific disaster preparedness policies to enhance resilience.<br>5. Successful community-based disaster management involves the full participation of not just the community, but also the government and private sector. This suggests that a multi-stakeholder approach is necessary for effective disaster preparedness.<br>6. Disaster preparedness in rural communities, such as the Orang Asli families, is currently low.<br>7. Both positive and negative experiences have been encountered by these communities during disasters, indicating varied levels of resilience and preparedness.
19.	Bogdan et al. (2021)	General hazard	Community groups	Surveys based on Statistics Canada's Survey of Emergency Preparedness and Resilience)	1. A very high percentage of participants in the Town of High River workshops (90.9% and 100%) experienced a major emergency or disaster in their lifetime.<br>2. The We're Ready! workshop was effective in improving participants' knowledge and skills related to emergency preparedness and resilience.<br>3. Participants reported increased motivation to enhance their emergency preparedness and strengthen their social ties.

					4. The workshop activities were generally well-received by participants.
20.	Ryan et al. (2020)	General hazard	Households and communities	Systematic review	1. Face-to-face techniques were generally more effective than mass media campaigns in promoting preparedness behaviors.<br>2. Engaging community members in the planning and implementation of preparedness activities can increase their sense of ownership and investment in the process.<br>3. The quality of reporting in the studies varied widely, making it difficult to assess the true effectiveness of some interventions.<br>4. There is a need for more rigorous evaluation of community engagement interventions for disaster preparedness.
21.	Wells et al. (2013)	General hazard	Households and communities	Surveys and interviews	Participants noted that their agencies provided education and outreach, but few described building two-way capacity in communities. There is need for development of a culturally relevant community engagement toolkit to build resilience at the community level.
22.	Eisenman et al. (2009)	General hazard	Households and communities	A survey to assess the water and food supplies in the household	1. Participants in both the platica and media groups reported significantly increased preparedness from pre-intervention to post-intervention, with a greater proportion of both arms reporting stockpiled water, food, radio, batteries, flashlights, first-aid kits, pet food, blankets, rain gear, cash, and written family communication plans.<br>2. The platica group had larger improvements in preparedness than did the media group for several key items.<br>3. The study demonstrated the value of respondent-driven sampling for recruiting hard-to-reach populations for research.
23.	Ardalan et al. (2013)	Flood, earthquake	Households	Questionnaire that assessed disaster awareness and readiness.	1. The intervention group had significantly higher disaster awareness and readiness scores compared to the control group.<br>2. The intervention was effective in improving specific aspects of disaster preparedness, including knowledge of emergency phone numbers, the importance of securing furniture during an earthquake, and the need for an emergency kit.

					3. The intervention was more effective in improving disaster awareness and readiness among households with lower levels of education and income.
24.	Oza et al. (2023)	COVID-19	Households and communities	Developed and implemented intervention without assessing its effectiveness	1. Community-based Outbreak Investigation and Response (COIR) can help enhance preparedness, public health capacity, and equity during the COVID-19 pandemic by developing and scaling up a local public health strategy that can conduct disease surveillance more effectively and respond proactively to local public health crises.<br>2. COIR involves a range of investigative and response functions, including local monitoring and investigation, coordination across interventions, and community engagement.<br>3. COIR can help local public health entities conduct disease surveillance more effectively, take a more proactive and efficient approach to mitigating transmission, coordinate response efforts, build community trust, and advance equity.
25.	Jauhar et al. (2021)	COVID-19	Community groups	Questionnaire of COVID-19 transmission risk (based on the Indonesian Ministry of Health)	1. Lack of knowledge about COVID-19 among the public, including the meaning of COVID-19, causes of COVID-19, and how COVID-19 is transmitted.<br>2. Health behaviors tend to be at risk, including lack of hand washing facilities, not knowing and practicing proper hand washing techniques, not wearing masks, not using masks when leaving the house, still having dental check-ups at the clinic, and still having meetings with more than 10 people.<br>3. Empowerment and community cooperation with health services are expected to be able to prevent COVID-19 transmission.
26.	Banerjee & Nair (2020)	COVID-19	Community groups	Developed and implemented intervention without assessing its effectiveness	Recommend the use of the proposed community-based toolkit for psycho-social management and preparedness to handle the COVID-19 pandemic

27.	Cvetković et al. (2020)	COVID-19	Individual	A structured questionnaire on knowledge, preparedness, and risk perception of the general population regarding COVID-19	1. The general population in Serbia has a moderate level of knowledge about COVID-19.<br>2. The general population in Serbia has a high level of risk perception regarding COVID-19.<br>3. The general population in Serbia has taken several preventive measures to protect themselves from COVID-19.<br>4. The main sources of information on COVID-19 for the general population in Serbia are television, scientific journals, and websites of local medical institutions.<br>5. The level of education is associated with some preventive behaviors, such as avoiding contacts with pets, shaking hands, maintaining recommended social distances, and storing more than a month's worth of food supplies in their homes.
28.	Musaazi et al. (2022)	Ebola	Households	A survey KAP questionnaire adapted Ebola Virus Disease	1. Only 4% of respondents had comprehensive knowledge of Ebola virus disease (EVD), and only 4% reported practicing all recommended prevention measures.<br>2. Factors associated with comprehensive knowledge and prevention practices included education level, exposure to EVD prevention messages, and living in a high-risk district.<br>3. Mass media was found to be effective in raising awareness and knowledge about EVD, but more targeted community engagement is needed to improve attitudes and behaviors related to prevention.
29.	Ching et al. (2023)	Flood	Individual	A modified pre-tested structured questionnaire from the CDC	1. Female participants, especially those older and blue-collar, generally had a better approach towards flood preparedness.<br>2. Among the male population, those with lower household income and those from the blue and white collared background were the determinants of good practice toward the flood.<br>3. Policymakers should pay attention to the male population during future flood prevention education programs.<br>4. Individual preparedness against flash floods is crucial in disaster management.

					5. Socio-economic conditions can lead to different outcomes, with the most vulnerable groups suffering the most.<br>6. Individuals with low-income in flood-prone areas are at higher risk due to limited access to recovery support mechanisms.<br>7. It's important to assess perception differences between males and females for better flood-related disaster preparedness.
30.	Samat et al. (2020)	Flood	Households	Face-to-face interview with a questionnaire evaluation based on Livelihood Vulnerability Index (LVI)	1. The Livelihood Vulnerability Index (LVI) is an effective tool for assessing the vulnerability of communities to floods.<br>2. The LVI and LVI-Intergovernmental Panel on Climate Change helped identify the basic vulnerability of these communities to floods and climate change.<br>3. All three areas are highly vulnerable to food shortages during floods, while water shows the lowest vulnerability index.<br>4. There is high sensitivity in terms of vulnerability in food, health, land, and financial aspects.<br>5. The key factors that contribute to the vulnerability of communities to floods in Pahang State are exposure, sensitivity, adaptive capacity, and livelihood strategies.<br>6. The measures taken by the government and local communities to reduce the impact of floods in the region have been largely ineffective.<br>7. There is a need for policymakers, government agencies, and local communities to work together to develop new strategies and find practical solutions to reduce people's vulnerability to floods in the region.
31.	Mhd Noor et al. (2022)	Flood	Community groups	A validated questionnaire that assessed knowledge, skills, and	1. The health education-based intervention (HEBI) intervention was effective in increasing knowledge, skills, and preparedness related to flood disaster management among the intervention group compared to the control group.<br>2. The HEBI intervention was associated with significant improvements in several factors related to flood disaster preparedness, including perceived susceptibility,

				preparedness related to flood disaster management	perceived severity, perceived benefits, and self-efficacy.<br>3. The HEBI intervention was more effective among participants who had lower baseline levels of knowledge, skills, and preparedness related to flood disaster management.
32.	Shariff & Hamidi (2019)	Flood	Community groups	Semi-structured interviews	1. A community-based approach for flood preparedness plan depends on: (1) flood frequency; (2) flood severity; (3) community awareness regarding flood hazard – knowledge and experience – that turn into willingness or volunteerism; and (4) types of social unit – preparedness plan for the whole village, which might be slightly different from that of a family or an individual.<br>2. Flood preparedness plan must meet specific objectives, which are: 1) completing not competing, (2) bridging the gap not redundancy and (3) a holistic approach not ad hoc basis.<br>3. Preparedness plans must meet basic guidelines and align with standard operating procedures and emergency response plans.<br>4. Key lessons include identifying key actors in disaster assistance and understanding the strengths and weaknesses of the villages through mapping.<br>5. Integrating indigenous and local knowledge with science can help develop resilient communities.<br>6. Communities themselves can become the first responders to disasters.<br>7. High technology is ineffective if individuals lack the knowledge to use it for their own safety.<br>8. Building a resilient community means equipping the community with knowledge about their own vulnerabilities and capabilities, through learning, experience, and traditional wisdom.
33.	Wan Mohammad et al. (2020)	Flood-related	Community groups	A validated questionnaire on	1. The baseline knowledge, attitude and practice (KAP) scores of the community regarding flood-related communicable diseases were low.

		outbreak		flood-related communicable diseases	2. The health education intervention was effective in improving the KAP scores of the community.<br>3. The coping mechanisms and experiences of the community pertaining to the 2014 flood disaster were identified:<br>-The community relied on various coping mechanisms, such as seeking help from neighbors, relatives, and friends, and relying on government aid and donations. The study also found that the community faced various challenges in coping with the flood disaster, such as lack of access to clean water and sanitation facilities, loss of property and livelihoods, and psychological distress.
34.	Stein-Zamir et al. (2019)	Measles	Community groups	Developed and implemented intervention without assessing its effectiveness	The tailored immunization program implemented by the Jerusalem District Health Office led to a significant increase in immunization coverage rates and a decrease in the incidence of measles in the population
35.	Watkins et al. (2019)	Mass casualty incidents	Hospital	Pre- and post-exercise surveys	1. 97% of participants reported that the simulation exercise was valuable and recommended additional simulations.<br>2. Participants demonstrated significant increases in self-confidence, skills, and knowledge ($p<.001$).<br>3. Self-confidence showed an increase of 0.8 (95%CI: 0.6-0.9), while both skills and knowledge improved by 0.4 (95%CI: 0.2-0.5).<br>4. The most substantial improvement was observed in self-confidence.
36.	Kang et al. (2022)	Healthcare-associated infection	Hospital	Literature review	1. Simulation-based training (SBT) remains underused in infection prevention despite its potential as a valuable supplement to traditional educational tools.<br>2. SBT has a wide reach and applicability, catering to learners across various levels, including students, nurses, physicians, and ancillary staff.

					3. SBT has the capability to improve learners' sense of competence and confidence in handling infection prevention measures.
37.	Zavaleta et al. (2018)	General disaster	Community	Narrative review	1. Community simulation training, reflecting whole community disaster resilience models, enhances communication, teamwork, self-care skills, and broadens understanding of community threats for better preparation and response.<br>2. Simulation-based education can accommodate a wide range of learners, spanning from high school students to medical professionals and public health officials, enabling inclusive participation and skill advancement across different proficiency levels and educational domains.<br>3. Disaster event simulations provide a safe learning environment that ensures physical, psychological, and contextual safety for participants, allowing individuals to engage, learn from failures, and explore various emergency response scenarios beyond their typical roles.<br>4. Debriefing of simulation exercises significantly aids learning by emphasizing that learners derive knowledge not just from experiences but mainly from reflecting on them.<br>5. Simulation is an extremely creative and flexible approach, drawing on the disparate disciplines of theatre, adult education, and clinical science.<br>6. Simulation facilitates the development of specific skills in cultural competence by incorporating diverse scenarios.
38.	Toyoda & Tanwattana (2023)	General disaster	Community	Inspiration cards and a "true-or-false" game element (facilitator-guided)	1. Gamification is a more effective alternative to conventional methods for extracting local knowledge for disaster risk management (DRM).<br>2. The game was found to be more effective than focus group discussions (FGDs) in terms of promoting adaptation and communication among participants.<br>3. The game extracted more knowledge of fact, usage, and value than FGDs. Techniques for extracting local knowledge, such as ethnographic methods including FGDs and key informant interviews

					4. The game was found to increase participants' willingness to cooperate with others and participate more actively in the game.<br>5. The game was found to be an effective tool for extracting detailed knowledge, especially in communication, to understand local contexts.<br>6. The study provides insights into the potential of gamification techniques for extracting local knowledge in other disaster-prone areas around the world.
39.	Maresch & Kampman (2022)	General disaster	Individual	Online Board Game	1. Game Engagement: Enjoyment of game mechanics, Triggered deeper conversations<br>2. Psychological Safety: Felt safe throughout, Online environment promoted safety and openness<br>3. Resource Expansion: Acquired new coping skills, Increased awareness of personal strengths and connections<br>4. Positive Impact: Experienced joy, gratitude, hope, Enhanced optimism and resilience
40.	Suppan et al. (2020)	COVID-19 pandemic	Individual	Free-to-play web-based serious game	1. The serious game "Escape COVID-19" was effective in increasing long-term care facility employees' willingness to adopt COVID-19 prevention and control practices.<br>2. Participants who played the serious game were more likely to report a willingness to change their infection prevention control (IPC) practices compared to those who received the control material.<br>3. The serious game was particularly effective in increasing participants' understanding of the reasons underlying the recommended IPC practices and their feeling of having an important role in the common effort against the epidemic.<br>4. Participants in the control group exhibited a higher intensity in their willingness to adopt the recommended behaviors, but the effect was not as strong as in the serious game group.<br>5. The serious game was well-received by participants, who reported that it was engaging, informative, and relevant to their work.<br>6. The study provides evidence for the effectiveness of serious games as a tool for

					educating healthcare professionals about infection prevention and control practices, particularly in the context of the COVID-19 pandemic.
41.	Krath et al. (2021)	General disaster	Individual, community and institutional	Systematic review on the applied theories	1. The most popular one is self-determination theory.<br>2. Gamification and serious games also increase self-efficacy, e.g., for reacting in emergencies, identifying cyber-security threads and performing learning tasks.<br>3. Related to self-efficacy theory, social comparison theory emphasizes the natural urge to assess oneself in comparison with others.
42.	Smith et al. (2020)	Infectious disease outbreak	Institutional	Serious games for serious crises: reflections from an infectious disease outbreak matrix game	1. The game was useful for foreign policy planning and for thinking about an international response to a global health crisis.<br>2. The matrix game format was reflective of decision-making contexts and emphasized the importance of thinking on your feet.<br>3. The progress of events in the game mirrored real interactions, including tugs-of-war over funding and tensions between regions.<br>4. The game prompted critical reflection on the importance of gender-based analysis in infectious disease outbreak preparedness and response.<br>5. Respondents recognized that if greater cooperation had been achieved during the preparedness conference, the response to the epidemic would have been more effective.
43.	Solinska-Nowak et al. (2018)	General disaster	Individual, community and institutional	Systematic review of serious games	1. The study analyzed 45 non-commercial digital and analog gaming activities related to DRM, identifying their characteristics, target groups, portrayed hazards, and possible DRM skills development.<br>2. The study found that serious games can be effective tools for DRM education and engagement, particularly for affected communities.<br>3. The study identified common objectives for DRM-related serious games, such as raising awareness, training decision-making, and improving collaboration.<br>4. The study found that serious games can be used to develop specific DRM-related skills, such as risk assessment, communication, and decision-making.

					5. The study noted that there is a lack of literature and research on the potential effectiveness of serious games in meeting their intended objectives.<br>6. The study found that the large number of games chosen for the analysis prevented more detailed content analysis, and that the authors often relied on information provided by the games' developers rather than direct exposure to the games.
44.	Findlay (2017)	General disaster	Individual	Review of digital games for various platforms such as desktop computers, mobile devices, and virtual reality	1. Digital games can be an effective tool for disaster education, as they can increase motivation and engagement among users.<br>2. The most effective disaster education games are those that incorporate key areas of disaster knowledge, such as hazard awareness, emergency planning, and emotional management.<br>3. There is a need for greater coordination and alignment of disaster education messages across jurisdictions and organizations.<br>4. "Stealth learning" methods, which bring educational content in an enjoyable form that does not look like typical educational material, can be effective in disaster education.<br>5. The Knowledge Framework developed in the study can be a useful tool for evaluating the effectiveness of disaster education games.<br>6. There is a need for further research on the effectiveness of disaster education games, particularly in terms of their impact on behavior change and long-term learning outcomes.
45.	Mossoux et al. (2016)	General disaster	Community	Board game or card game (facilitator-guided)	1. Hazagora helps develop important social and negotiation skills.<br>2. Discussions among people who would not sit down together or otherwise interact when using traditional teaching methods lead to a better sharing of knowledge and experience.<br>3. Each game session is unique, and the extent of the impacts on the board game will differ, which may result in more attention being given to one or another disaster factor.<br>4. A statistically significant improvement is observed regarding the players'

					understanding of the importance of land use spatial planning, community strategies, and home adaptation to develop a resilient community.<br>5. When asked whether settlement location is mainly controlled by the will to avoid hazards, players initially answer negatively but seem to agree more with this statement after the game.

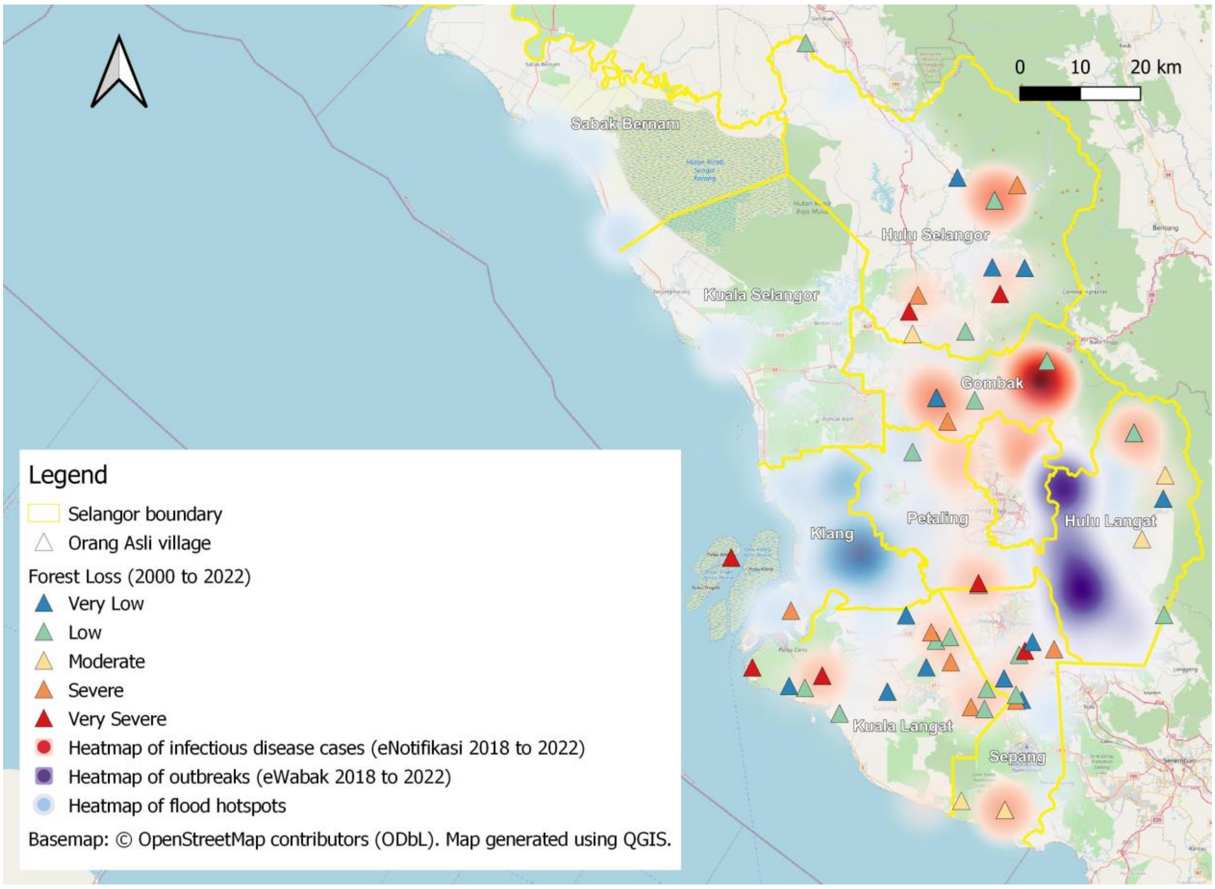

S2 Fig. Mapping of Orang Asli vulnerability in Selangor, displaying village locations in areas with forest loss overlaid with heatmaps of infectious disease notifications from eNotifikasi and outbreak data from eWabak. Map data from OpenStreetMap.

References

Ardalan, A., Mowafi, H., Malekafzali Ardakani, H., Abolhasanai, F., Zanganeh, A. M., Safizadeh, H., Salari, S., & Zonoobi, V. (2013). Effectiveness of a primary health care program on urban and rural community disaster preparedness, islamic republic of Iran: A community intervention trial. *Disaster Medicine and Public Health Preparedness*, *7*(5), 481–490. https://doi.org/10.1017/dmp.2013.93

Banerjee, D., & Nair, V. S. (2020). Handling the COVID-19 pandemic: Proposing a community based toolkit for psycho-social management and preparedness. In *Asian Journal of Psychiatry* (Vol. 51). Elsevier B.V. https://doi.org/10.1016/j.ajp.2020.102152

Barbier, E. B. (2021). Habitat loss and the risk of disease outbreak. *Journal of Environmental Economics and Management*, *108*. https://doi.org/10.1016/j.jeem.2021.102451

Bisan, S., & Goh, I. (2015). *A Scoping Study : A Focus on Indigenous Communities MALAYSIA Asian-Pacific Resource and Research Centre for Women (ARROW)*.

Bogdan, E. (Evalyna) A., Roszko, A. M., Beckie, M. A., & Conway, A. (2021). We're ready! Effectiveness of community disaster preparedness workshops across different community groups in Alberta, Canada. *International Journal of Disaster Risk Reduction*, *55*, 102060. https://doi.org/10.1016/j.ijdrr.2021.102060

Ching, S. M., Wei, L. K., Maharajan, M. K., Salim, H., Ying, N. J., Yean, N. K., Rashid, A. A., Sivaratnam, D. A. / P., Shamsudin, N. H., Ahmad, I., & Ramachandran, V. (2023). Risk Perception and Emergency Preparedness Against Flood Affected Participants From the Primary Health Care Centre, Malaysia: A Comparison Between Genders. *Malaysian Journal of Medicine and Health Sciences*, *19*(4), 8–14. https://doi.org/10.47836/mjmhs19.4.3

Chinnasamy, S., Rosnon, M. R., & Gill, Sarjit. S. (2021). Crisis Communication Relief Management and Preparedness: Covid-19 Disease Outbreak for Indigenous Peoples in Malaysia. *International Journal of Academic Research in Business and Social Sciences*, *11*(11). https://doi.org/10.6007/ijarbss/v11-i11/11770

Cvetković, V. M., Nikolić, N., Nenadić, U. R., Öcal, A., Noji, E. K., & Zečević, M. (2020). Preparedness and preventive behaviors for a pandemic disaster caused by COVID-19 in Serbia. *International Journal of Environmental Research and Public Health*, *17*(11), 1–23. https://doi.org/10.3390/ijerph17114124

Dorasamy, M., Raman, M., Muthaiyah, S., Kaliannan, M., & Alam, S. (2010, March). Disaster Preparedness in Malaysia: An Exploratory Study. *Proceedings of 4th WSEAS Marketing and Management Conference*. http://www.mmu.edu.myhttp://www.mmu.edu.myhttp://www.mmu.edu.myhttp://www.uitm.edu.my

Eisenman, D. P., Glik, D., Gonzalez, L., Maranon, R., Zhou, Q., Tseng, C.-H., & Asch, S. M. (2009). Improving Latino Disaster Preparedness Using Social Networks. *American Journal of Preventive Medicine*, *37*(6), 512–517. https://doi.org/10.1016/j.amepre.2009.07.022

Fahmi, A., Yusof, M., & Wook, I. (2020). Indigenous Peoples Living In Protected Areas: An Observation On The Impact Of Covid-19 In Kampung Peta, Endau-Rompin National Park. *INSLA E-Proceedings*, *3*(1), 44–49. www.insla.usim.edu.my

Findlay, B. (2017). *Digital Games in Disaster Preparedness Education*. https://doi.org/10.13140/RG.2.2.24698.03527

Hawkes, F. M., Manin, B. O., Cooper, A., Daim, S., Homathevi, R., Jelip, J., Husin, T., & Chua, T. H. (2019). Vector compositions change across forested to deforested ecotones in emerging areas of zoonotic malaria transmission in Malaysia. *Scientific Reports*, *9*(1). https://doi.org/10.1038/s41598-019-49842-2

Idrus, R., Man, Z., Williams-Hunt, A., & Chopil, T. Y. (2021). Indigenous resilience and the COVID-19 response: a situation report on the Orang Asli in Peninsular Malaysia. *AlterNative*, *17*(3), 439–443. https://doi.org/10.1177/11771801211038723

Jauhar, M., Rasdiyanah, R., Kusumawardani, L. H., Rachmawati, U., & Ayu Putu Desy Rohana, I. G. (2021). Community based intervention: Local village preparedness in prevention and control of COVID-19. *Journal of Community Empowerment for Health*, *4*(3), 203. https://doi.org/10.22146/jcoemph.63934

Kang, M., Nagaraj, M. B., Campbell, K. K., Nazareno, I. A., Scott, D. J., Arocha, D., & Trivedi, J. B. (2022). The role of simulation-based training in healthcare-associated infection (HAI) prevention. *Antimicrobial Stewardship and Healthcare Epidemiology*, *2*(1). https://doi.org/10.1017/ash.2021.257

Krath, J., Schürmann, L., & von Korflesch, H. F. O. (2021). Revealing the theoretical basis of gamification: A systematic review and analysis of theory in research on gamification, serious games and game-based learning. *Computers in Human Behavior*, *125*. https://doi.org/10.1016/j.chb.2021.106963

Liew, J. W. K., Mahpot, R. B., Dzul, S., Razak, H. A. B. A., Azizi, N. A. B. A. S., Kamarudin, M. B., Russell, B., Lim, K. L., De Silva, J. R., Lim, B. S., Jelip, J., Mudin, R. N. B., & Lau, Y. L. (2018). Importance of proactive malaria case surveillance and management in Malaysia. *American Journal of Tropical Medicine and Hygiene*, *98*(6), 1709–1713. https://doi.org/10.4269/ajtmh.17-1010

Maresch, I., & Kampman, H. (2022). Playing for Resilience in a Pandemic; Exploring the Role of an Online Board Game in Recognising Resources. *International Journal of Applied Positive Psychology*. https://doi.org/10.1007/s41042-022-00069-z

Mhd Noor, M. T., Kadir Shahar, H., Baharudin, M. R., Syed Ismail, S. N., Abdul Manaf, R., Md Said, S., Ahmad, J., & Muthiah, S. G. (2022). Facing flood disaster: A cluster

randomized trial assessing communities' knowledge, skills and preparedness utilizing a health model intervention. *PloS One*, *17*(11), e0271258. https://doi.org/10.1371/journal.pone.0271258

Mohd Radi, M. F., Hashim, J. H., Jaafar, M. H., Hod, R., Ahmad, N., Nawi, A. M., Baloch, G. M., Ismail, R., & Ayub, N. I. F. (2018). Leptospirosis outbreak after the 2014 major flooding event in Kelantan, Malaysia: A spatial-temporal analysis. *American Journal of Tropical Medicine and Hygiene*, *98*(5), 1281–1295. https://doi.org/10.4269/ajtmh.16-0922

Mohd Rosman, M. H. W., Yong, C. L., Azman, M. U., & Mohd Ishar, M. I. (2020). The Health Issue in Orang Asli Community. *Malaysian Journal of Social Sciences and Humanities (MJSSH)*, *5*(2), 36–41. https://doi.org/10.47405/mjssh.v5i2.360

Morand, S., & Lajaunie, C. (2021). Outbreaks of Vector-Borne and Zoonotic Diseases Are Associated With Changes in Forest Cover and Oil Palm Expansion at Global Scale. *Frontiers in Veterinary Science*, *8*. https://doi.org/10.3389/fvets.2021.661063

Mossoux, S., Delcamp, A., Poppe, S., Michellier, C., Canters, F., & Kervyn, M. (2016). Hazagora: Will you survive the next disaster?-A serious game to raise awareness about geohazards and disaster risk reduction. *Natural Hazards and Earth System Sciences*, *16*(1), 135–147. https://doi.org/10.5194/nhess-16-135-2016

Musaazi, J., Namageyo-Funa, A., Carter, V. M., Carter, R. J., Lamorde, M., Apondi, R., Bakyaita, T., Boore, A. L., Brown, V. R., Homsy, J., Kigozi, J., Koyuncu, A., Nabaggala, M. S., Nakate, V., Nkurunziza, E., Stowell, D. F., Walwema, R., Olowo, A., & Jalloh, M. F. (2022). Evaluation of Community Perceptions and Prevention Practices Related to Ebola Virus as Part of Outbreak Preparedness in Uganda, 2020. *Global Health: Science and Practice*, *10*(3), 1–15. www.ghspjournal.org

Nungsari, M., Hui Yin, C., Fong, N., & Pillai, V. (2021). Understanding the impact of the COVID-19 outbreak on vulnerable populations in Malaysia through an ethical lens: A study of NGOs and organizations involved in aid distribution. *Wellcome Open Research*, *6*, 263. https://doi.org/10.12688/wellcomeopenres.17239.1

Ochta Pebriyanti, D., Endro Sulistyono, R., & Mahardhika Rahmawati, P. (2023). ROLE COMMUNITY-BASED DISASTER MANAGEMENT SYSTEM: SYSTEMATIC REVIEW. *Indonesia Proceeding International Agronursing Conference*, *409*(1), 2023.

Oza, S., Chen, F., Selser, V., Clougherty, M. M., Dale, K. D., Johnson, J. I., Brock-Fisher, T., Seung, K. J., & Bourdeaux, M. (2023). Community-Based Outbreak Investigation And Response: Enhancing Preparedness, Public Health Capacity, And Equity. *Health Affairs*, *42*(3), 349–356. https://doi.org/10.1377/hlthaff.2022.01257

Rosnon, M. R., Zuhairi, M. H., Abdul Razak, M. A., Jalaludin, M. L., & Ibrahim, R. (2022). Knowledge, Attitudes and Practice Towards Covid-19 Among the Orang Asli Elderly in Peninsular Malaysia. *International Journal of Academic Research in Business and Social Sciences*, *12*(10). https://doi.org/10.6007/ijarbss/v12-i10/15208

Ryan, B., Johnston, K. A., Taylor, M., & McAndrew, R. (2020). Community engagement for disaster preparedness: A systematic literature review. *International Journal of Disaster Risk Reduction*, *49*. https://doi.org/10.1016/j.ijdrr.2020.101655

Sadeka, S., Mohamad, M. S., & Sarkar, M. S. K. (2020). Disaster experiences and preparedness of the Orang Asli Families in Tasik Chini of Malaysia: A conceptual framework towards building disaster resilient community. *Progress in Disaster Science*, *6*. https://doi.org/10.1016/j.pdisas.2020.100070

Sadeka, S., Mohamad, M. S., Sarkar, M. S. K., & Al-Amin, A. Q. (2020). Conceptual Framework and Linkage Between Social Capital and Disaster Preparedness: A Case of Orang Asli Families in Malaysia. *Social Indicators Research*, *150*(2), 479–499. https://doi.org/10.1007/s11205-020-02307-w

Samat, A. H. A., Rashid, A. A., Yunus, N. A. M., Salim, A. M. H., & Musa, H. (2020). A Malaysian medical non-governmental organisation's (NGO) experience in the emergency response for COVID-19 using the 'whole-of-society collaborative' concept. *Disaster Medicine and Public Health Preparedness*, 1–13. https://doi.org/10.1017/dmp.2021.106

Shafii, N. Z., Saudi, A. S. M., Pang, J. C., Abu, I. F., Sapawe, N., Kamarudin, M. K. A., & Mohamad, M. H. N. (2023). Association of Flood Risk Patterns with Waterborne Bacterial Diseases in Malaysia. *Water (Switzerland)*, *15*(11). https://doi.org/10.3390/w15112121

Shariff, N. N. M., & Hamidi, Z. S. (2019). Community-based approach for a flood preparedness plan in Malaysia. *Jàmbá: Journal of Disaster Risk Studies*, *11*(1). https://doi.org/10.4102/jamba.v11i1.598

Smith, J., Sears, N., Taylor, B., & Johnson, M. (2020). Serious games for serious crises: Reflections from an infectious disease outbreak matrix game. *Globalization and Health*, *16*(1). https://doi.org/10.1186/s12992-020-00547-6

Solinska-Nowak, A., Magnuszewski, P., Curl, M., French, A., Keating, A., Mochizuki, J., Liu, W., Mechler, R., Kulakowska, M., & Jarzabek, L. (2018). An overview of serious games for disaster risk management – Prospects and limitations for informing actions to arrest increasing risk. In *International Journal of Disaster Risk Reduction* (Vol. 31, pp. 1013–1029). Elsevier Ltd. https://doi.org/10.1016/j.ijdrr.2018.09.001

Stein-Zamir, C., Abramson, N., Edelstein, N., Shoob, H., Zentner, G., & Zimmerman, D. R. (2019). Community-Oriented Epidemic Preparedness and Response to the Jerusalem 2018–2019 Measles Epidemic. *American Journal of Public Health*, *109*(12), 1714–1716. https://doi.org/10.2105/AJPH.2019.305343

Suppan, M., Gartner, B., Golay, E., Stuby, L., White, M., Cottet, P., Abbas, M., Iten, A., Harbarth, S., & Suppan, L. (2020). Teaching Adequate Prehospital Use of Personal Protective Equipment During the COVID-19 Pandemic: Development of a Gamified e-Learning Module. *JMIR Serious Games*, *8*(2), e20173. https://doi.org/10.2196/20173

Toyoda, Y., & Tanwattana, P. (2023). Extracting local disaster knowledge through gamification in a flood management model community in Thailand. *Progress in Disaster Science*, *20*, 100294. https://doi.org/10.1016/j.pdisas.2023.100294

Venugopalan, B., R Nik Rubiah, N. A., Meftahuddin, T., Ayu, M., Prema, R., Ruhaini, I., & Murugan, S. (2004). Hepatitis A Outbreak in Hulu Langat District, Selangor State, Malaysia During April - October 2002. *Med J Malaysia*, *59*(5), 670–673.

Wan Mohammad, M. Z., Wan Mansor, N. A., Aman Hamid, N. A., Sukeri, S., Hassan, H., Mohamed, Z., Yeong Yeh, L., Muhd Besari, A., Draman, N., & Zakaria, R. (2020). Effectiveness of Community-Based Health Education on Preparedness for Flood-Related Communicable Diseases in Kelantan. *Malaysian Journal of Public Health Medicine*, *2020*(3), 117–124.

Watkins, N., Johnston, A. N., McNamee, P., Muter, N., Huang, C., ling Li, Y., Samsuddin, A., & Sweeny, A. (2019). Preparing for Mass Casualties: Improving Staff Preparedness and Hospital Operations through Multidisciplinary Simulation Training in Disaster Management. *Prehospital and Disaster Medicine*, *34*(s1), s81–s82. https://doi.org/10.1017/s1049023x19001717

Wells, K. B., Tang, J., Lizaola, E., Jones, F., Brown, A., Stayton, A., Williams, M., Chandra, A., Eisenman, D., Fogleman, S., & Plough, A. (2013). Applying community engagement to disaster planning: Developing the vision and design for the Los Angeles county community disaster resilience initiative. *American Journal of Public Health*, *103*(7), 1172–1180. https://doi.org/10.2105/AJPH.2013.301407

Zavaleta, K. W., Asirvatham, U., Callies, B., Franz, W. B., Scanlan-Hanson, L., & Molella, R. G. (2018). Improving Community Resilience through Disaster Simulation: How Simulation-based Education Augments Emergency Management Exercises. *Journal of Homeland Security and Emergency Management*, *15*(2). https://doi.org/10.1515/jhsem-2018-0002
