# Supplementary material for: Development of X-SIAGA: A disease X and outbreak preparedness intervention for indigenous households in Selangor, Malaysia
Source: PLoS One. 2026 Mar 30;21(3):e0345785. doi: 10.1371/journal.pone.0345785 (PMC13035154; doi:10.1371/journal.pone.0345785)
Supplement: S3 File — Summary of content and face validation for the X-SIAGA intervention and HOPE questionnaire. (PDF) [file pone.0345785.s003.pdf]

S3 File. Content and face validation results.

S3.1 Table. Sociodemographic backgrounds of the experts in content validation (n=5).

**Expert**	**Age**	**Ethnicity**	**Education level**	**Field of expertise**	**Years of experience**
1	50	Malay	Tertiary education (Masters)	Orang Asli behavioral research	22
2	54	Orang Asli	Tertiary education (Masters)	Public health	24
3	41	Malay	Tertiary education (PhD)	Veterinary science	8
4	36	Malay	Secondary school	Orang Asli community affairs	12
5	33	Malay	Tertiary education (Bachelors)	Orang Asli community affairs	3

S3.2 Table. Relevance to intervention objectives ratings of the X-SIAGA materials by expert panel (n=5).

X-SIAGA materials	Experts						Experts in agreement	I-CVI	UA
	1	2	3	4	5				
1. Lecture: What is Disease X?	1	1	1	1	1		5	1	1
2. Lecture: What is an outbreak?	1	1	1	1	1		5	1	1
3. Lecture: When can Disease X emerge and spread into an outbreak?	1	1	1	1	1		5	1	1
4. Lecture: Where can Disease X and outbreaks spread?	1	1	1	1	1		5	1	1
5. Lecture: How can Disease X and outbreaks spread?	1	1	1	1	1		5	1	1
6. Video session: How can Disease X and outbreaks spread?	1	1	1	1	1		5	1	1
7. Lecture: Who is at higher risk of Disease X and outbreaks?	1	1	1	1	1		5	1	1
8. Lecture: Why should we be concerned about Disease X?	1	1	1	1	1		5	1	1
9. Lecture: What is meant by "household preparedness for Disease X and outbreaks"?	1	1	1	1	1		5	1	1
10. Lecture: What is the importance of household preparedness for Disease X and outbreaks?	1	1	1	1	1		5	1	1
11. Lecture: Why are Orang Asli communities at risk of Disease X and outbreaks?	1	1	1	1	1		5	1	1
12. Lecture: Who is responsible for taking preparedness actions?	1	1	1	1	1		5	1	1
13. Lecture: How can households prepare for Disease X and outbreaks?	1	1	1	1	1		5	1	1
14. Video session: Understanding disease and outbreak spread and household preparedness (Nipah Virus Outbreak)	1	1	1	1	1		5	1	1
15. Video session: Understanding disease and outbreak spread and household preparedness (Ebola Virus Outbreak)	1	1	1	1	1		5	1	1
16. Video session: Understanding disease and outbreak spread and household preparedness (COVID-19 Pandemic)	1	1	1	1	1		5	1	1
17. Hands-on session: 7 steps of proper handwashing	1	1	1	1	1		5	1	1
18. Hands-on session: Developing household planning for Disease X and outbreaks using the template provided in the booklet	1	1	1	1	1		5	1	1

19. Hands-on session: Learning symptoms of potential Disease X and signs of outbreaks through puzzle activities	1	1	1	1	1		5	1	1
20. Hands-on session: Learning steps for handling suspected sick or dead animals and family members during Disease X or outbreaks through puzzle activities	1	1	1	1	1		5	1	1
21. Puzzle explanation: Handling sick or dead animals suspected of Disease X or outbreaks	1	1	1	1	1		5	1	1
22. Puzzle explanation: Handling family members suspected of having Disease X or outbreaks	1	1	1	1	1		5	1	1
23. Lecture: Cleaning and disinfecting the home and refilling the outbreak kit after the outbreak	1	1	1	1	1		5	1	1
24. Lecture: Hands-only CPR	1	1	1	1	1		5	1	1
25. Hands-on session: Hands-only CPR	1	1	1	1	1		5	1	1
26. Simulation exercise 1 (handling sick or dead animals during a suspected Disease X or outbreak)	1	1	1	1	1		5	1	1
27. Simulation exercise 2 (handling sick family members during a suspected Disease X or outbreak)	1	1	1	1	1		5	1	1
28. Simulation exercise 3 (performing hands-only CPR for life-saving measures)	1	1	1	1	1		5	1	1
29. Game-based learning: Wabak X instructional leaflet	1	1	1	1	1		5	1	1
30. Game-based learning: Wabak X game mechanics	1	1	1	1	1		5	1	1
31. Game-based learning: Lessons from Wabak X gameplay	1	1	1	1	1		5	1	1
32. Game-based learning: Wabak X illustrations	1	1	1	1	1		5	1	1
							**SCVI/Ave**	**1.0**	
**Proportion of relevance to intervention objectives**	1	1	1	1	1		**SCVI/UA**		**1.0**
	**Average of proportion of contents judged as relevant to intervention objectives across experts**					**1.0**			

S3.3 Table. Clarity ratings of the X-SIAGA materials by expert panel (n=5).

**X-SIAGA materials**	**Experts**						**Experts in agreement**	**I-CVI**	**UA**
	**1**	**2**	**3**	**4**	**5**				
1. Lecture: What is Disease X?	1	1	1	1	1		5	1	1
2. Lecture: What is an outbreak?	1	1	1	1	1		5	1	1
3. Lecture: When can Disease X emerge and spread into an outbreak?	1	1	1	1	1		5	1	1
4. Lecture: Where can Disease X and outbreaks spread?	1	1	1	1	1		5	1	1
5. Lecture: How can Disease X and outbreaks spread?	1	1	1	1	1		5	1	1
6. Video session: How can Disease X and outbreaks spread?	1	1	1	1	1		5	1	1
7. Lecture: Who is at higher risk of Disease X and outbreaks?	1	1	1	1	1		5	1	1
8. Lecture: Why should we be concerned about Disease X?	1	1	1	1	1		5	1	1
9. Lecture: What is meant by "household preparedness for Disease X and outbreaks"?	1	1	1	1	1		5	1	1
10. Lecture: What is the importance of household preparedness for Disease X and outbreaks?	1	1	1	1	1		5	1	1
11. Lecture: Why are Orang Asli communities at risk of Disease X and outbreaks?	1	1	1	1	1		5	1	1
12. Lecture: Who is responsible for taking preparedness actions?	1	1	1	1	1		5	1	1
13. Lecture: How can households prepare for Disease X and outbreaks?	1	1	1	1	1		5	1	1
14. Video session: Understanding disease and outbreak spread and household preparedness (Nipah Virus Outbreak)	1	1	1	1	1		5	1	1
15. Video session: Understanding disease and outbreak spread and household preparedness (Ebola Virus Outbreak)	1	1	1	1	1		5	1	1
16. Video session: Understanding disease and outbreak spread and household preparedness (COVID-19 Pandemic)	1	1	1	1	1		5	1	1
17. Hands-on session: 7 steps of proper handwashing	1	1	1	1	1		5	1	1
18. Hands-on session: Developing household planning for Disease X and outbreaks using the template provided in the booklet	1	1	1	1	1		5	1	1

19. Hands-on session: Learning symptoms of potential Disease X and signs of outbreaks through puzzle activities	1	1	1	1	1		5	1	1
20. Hands-on session: Learning steps for handling suspected sick or dead animals and family members during Disease X or outbreaks through puzzle activities	1	1	1	1	1		5	1	1
21. Puzzle explanation: Handling sick or dead animals suspected of Disease X or outbreaks	1	1	1	1	1		5	1	1
22. Puzzle explanation: Handling family members suspected of having Disease X or outbreaks	1	1	1	1	1		5	1	1
23. Lecture: Cleaning and disinfecting the home and refilling the outbreak kit after the outbreak	1	1	1	1	1		5	1	1
24. Lecture: Hands-only CPR	1	1	1	1	1		5	1	1
25. Hands-on session: Hands-only CPR	1	1	1	1	1		5	1	1
26. Simulation exercise 1 (handling sick or dead animals during a suspected Disease X or outbreak)	1	1	1	1	1		5	1	1
27. Simulation exercise 2 (handling sick family members during a suspected Disease X or outbreak)	1	1	1	1	1		5	1	1
28. Simulation exercise 3 (performing hands-only CPR for life-saving measures)	1	1	1	1	1		5	1	1
29. Game-based learning: Wabak X instructional leaflet	1	1	1	1	1		5	1	1
30. Game-based learning: Wabak X game mechanics	1	1	1	1	1		5	1	1
31. Game-based learning: Lessons from Wabak X gameplay	1	1	1	1	1		5	1	1
32. Game-based learning: Wabak X illustrations	1	1	1	1	1		5	1	1
							**SCVI/Ave**	**1.0**	
**Proportion of clarity**	1	1	1	1	1		**SCVI/UA**		**1.0**
	**Average of proportion of contents judged as clear across experts**					**1.0**			

S3.4 Table. Relevance to intervention objectives ratings of the HOPE items by expert panel (n=5).

HOPE items	Experts						Experts in agreement	I-CVI	UA
	1	2	3	4	5				
K1. Penyakit X adalah penyakit berjangkit yang baharu dan belum dikenalpasti	1	1	1	1	1		5	1	1
K2. Penyakit X adalah penyakit yang boleh terjadi di mana-mana sahaja di dunia	1	1	1	1	1		5	1	1
K3. Haiwan tidak mungkin menyebarkan Penyakit X kepada manusia	1	1	1	1	1		5	1	1
K4. Sawan adalah antara gejala jika kuman menyerang paru-paru dan sistem pernafasan	1	1	1	1	1		5	1	1
K5. Wabak disyaki berlaku apabila terdapat 2 atau lebih orang yang mengalami gejala sama, dalam tempoh masa yang hampir sama, dan mereka ada kaitan dengan tempat atau orang yang sama	1	1	1	1	1		5	1	1
K6. Wabak Penyakit X boleh diramalkan bila akan berlaku	1	1	1	1	1		5	1	1
K7. Wabak Penyakit X diramalkan lebih teruk dan merbahaya daripada COVID-19	1	1	1	1	1		5	1	1
K8. Hanya pihak berkuasa kesihatan sahaja yang boleh melaporkan wabak	1	1	1	1	1		5	1	1
K9. Persediaan isi rumah dalam menghadapi Penyakit X dan wabak bermaksud mengambil langkah untuk melindungi diri sendiri sahaja sebelum ianya berlaku	1	1	1	1	1		5	1	1
K10. Sebahagian persediaan isi rumah menghadapi Penyakit X dan wabak ialah menyimpan maklumat untuk mendapatkan bantuan jika Penyakit X atau wabak berlaku	1	1	1	1	1		5	1	1
K11. Sebahagian persediaan isi rumah untuk menghadapi Penyakit X dan wabak ialah merancang tindakan yang perlu diambil jika Penyakit X atau wabak berlaku	1	1	1	1	1		5	1	1
K12. Bekerja dengan haiwan dan produk haiwan (seperti daging, kulit, dan tanduk) boleh meningkatkan risiko Penyakit X dan wabak merebak dari haiwan kepada manusia	1	1	1	1	1		5	1	1

K13. Makan daging mentah atau daging haiwan liar boleh meningkatkan risiko Penyakit X dan wabak merebak dari haiwan kepada manusia	1	1	1	1	1		5	1	1
K14. Kawasan yang sesak mempunyai risiko yang lebih rendah untuk penyebaran Penyakit X dan wabak	1	1	1	1	1		5	1	1
K15. Orang dengan masalah kesihatan kronik (contoh: asma, kencing manis, barah) berisiko lebih tinggi untuk mengalami sakit teruk jika dijangkiti Penyakit X atau wabak	1	1	1	1	1		5	1	1
K16. Kanak-kanak di bawah 5 tahun berisiko lebih tinggi untuk mengalami sakit teruk jika dijangkiti Penyakit X atau wabak	1	1	1	1	1		5	1	1
A1. Saya dan ahli isi rumah saya berisiko dijangkiti Penyakit X dan wabak	1	1	1	1	1		5	1	1
A2. Saya dan ahli isi rumah saya berisiko menyebarkan Penyakit X dan wabak kepada orang lain	1	1	1	1	1		5	1	1
A3. Saya dan ahli isi rumah berisiko untuk meninggal dunia sekiranya dijangkiti Penyakit X dan wabak	1	1	1	1	1		5	1	1
A4. Penyakit X dan wabak berisiko menyebabkan tekanan jiwa atau masalah kesihatan mental kepada saya dan ahli isi rumah saya	1	1	1	1	1		5	1	1
A5. Penyakit X dan wabak tidak akan menjejaskan sumber pendapatan dan mata pencarian saya serta ahli isi rumah saya	1	1	1	1	1		5	1	1
A6. Tindakan persediaan dapat melindungi diri sendiri dan ahli isi rumah daripada jangkitan Penyakit X dan wabak	1	1	1	1	1		5	1	1
A7. Tindakan persediaan menghadapi Penyakit X dan wabak membantu saya dan ahli isi rumah mengetahui apa yang perlu dilakukan jika dijangkiti Penyakit X dan wabak	1	1	1	1	1		5	1	1
A8. Persediaan menghadapi Penyakit X dan wabak adalah tanggungjawab kerajaan sepenuhnya	1	1	1	1	1		5	1	1
A9. Saya dan ahli isi rumah berupaya mengambil langkah untuk bersiap sedia menghadapi Penyakit X dan wabak dengan sendiri	1	1	1	1	1		5	1	1

A10. Saya dan ahli isi rumah berpendapat, buat masa sekarang, persediaan menghadapi Penyakit X dan wabak adalah tidak penting	1	1	1	1	1		5	1	1
A11. Saya dan ahli isi rumah berpendapat, persediaan menghadapi Penyakit X dan wabak memerlukan belanja yang banyak	1	1	1	1	1		5	1	1
A12. Saya dan ahli isi rumah tidak suka mematuhi arahan daripada pihak berkuasa kesihatan seperti kuarantin, pakai pelitup muka, atau penjarakan fizikal, jika dijangkiti Penyakit X atau wabak	1	1	1	1	1		5	1	1
A13. Saya dan ahli isi rumah tidak suka menerima rawatan perubatan seperti diambil darah, makan ubat, atau dicucuk vaksin, jika dijangkiti Penyakit X atau wabak	1	1	1	1	1		5	1	1
A14. Saya dan ahli isi rumah tidak cukup masa untuk mendapatkan rawatan kesihatan jika terkena Penyakit X dan wabak	1	1	1	1	1		5	1	1
A15. Saya dan ahli isi rumah tidak cukup wang untuk mendapatkan rawatan kesihatan jika terkena Penyakit X dan wabak	1	1	1	1	1		5	1	1
A16. Saya dan ahli isi rumah tidak ada pengangkutan untuk mendapatkan rawatan kesihatan jika terkena Penyakit X dan wabak	1	1	1	1	1		5	1	1
P1. Saya dan ahli isi rumah telah menyediakan senarai nombor telefon perkhidmatan kecemasan dan kontak penting	1	1	1	1	1		5	1	1
P2. Saya dan ahli isi rumah telah menyediakan senarai maklumat kesihatan ahli isi rumah	1	1	1	1	1		5	1	1
P3. Saya dan ahli isi rumah telah mengenal pasti siapa untuk dihubungi apabila mengesyaki kemungkinan Penyakit X dan wabak	1	1	1	1	1		5	1	1
P4. Saya dan ahli isi rumah telah mengenal pasti cara untuk kami menerima maklumat berkaitan Penyakit X dan wabak	1	1	1	1	1		5	1	1
P5. Saya dan ahli isi rumah telah menyediakan bekalan makanan dan air cukup untuk sekurang-kurangnya 3 hari	1	1	1	1	1		5	1	1

P6. Saya dan ahli isi rumah telah memilih bilik atau ruang untuk pengasingan ahli rumah yang sakit dijangkiti Penyakit X atau wabak	1	1	1	1	1		5	1	1
P7. Saya dan ahli isi rumah telah menyediakan ubat demam di dalam kit wabak	1	1	1	1	1		5	1	1
P8. Saya dan ahli isi rumah telah menyediakan barangan penjagaan luka di dalam kit wabak	1	1	1	1	1		5	1	1
P9. Saya dan ahli isi rumah saya telah menyediakan barangan pembasmi kuman di dalam kit wabak	1	1	1	1	1		5	1	1
P10. Saya dan ahli isi rumah saya telah menyediakan peralatan perlindungan diri di dalam kit wabak	1	1	1	1	1		5	1	1
P11. Nomborkan mengikut turutan yang betul langkah-langkah cuci tangan menggunakan sabun atau sanitizer	1	1	1	1	1		5	1	1
P12. Nomborkan mengikut turutan yang betul langkah-langkah apabila terdapat ahli keluarga yang sakit tenat disyaki akibat jangkitan Penyakit X atau wabak	1	1	1	1	1		5	1	1
P13. Nomborkan mengikut turutan yang betul langkah-langkah apabila terdapat ahli keluarga yang tiba-tiba rebah dan tidak sedarkan diri	1	1	1	1	1		5	1	1
							**SCVI/Ave**	**1.0**	
**Proportion of relevance to intervention objectives**	1	1	1	1	1		**SCVI/UA**		**1.0**
	**Average of proportion of items judged as relevant to intervention objectives across experts**					**1.0**			

S3.5 Table. Clarity ratings of the HOPE items by expert panel (n=5).

**HOPE items**	**Experts**						**Experts in agreement**	**I-CVI**	**UA**
	**1**	**2**	**3**	**4**	**5**				
K1. Penyakit X adalah penyakit berjangkit yang baharu dan belum dikenalpasti	1	1	1	1	1		5	1	1
K2. Penyakit X adalah penyakit yang boleh terjadi di mana-mana sahaja di dunia	1	1	1	1	1		5	1	1
K3. Haiwan tidak mungkin menyebarkan Penyakit X kepada manusia	1	1	1	1	1		5	1	1
K4. Sawan adalah antara gejala jika kuman menyerang paru-paru dan sistem pernafasan	1	1	1	1	1		5	1	1
K5. Wabak disyaki berlaku apabila terdapat 2 atau lebih orang yang mengalami gejala sama, dalam tempoh masa yang hampir sama, dan mereka ada kaitan dengan tempat atau orang yang sama	1	1	1	1	1		5	1	1
K6. Wabak Penyakit X boleh diramalkan bila akan berlaku	1	1	1	1	1		5	1	1
K7. Wabak Penyakit X diramalkan lebih teruk dan merbahaya daripada COVID-19	1	1	1	1	1		5	1	1
K8. Hanya pihak berkuasa kesihatan sahaja yang boleh melaporkan wabak	1	1	1	1	1		5	1	1
K9. Persediaan isi rumah dalam menghadapi Penyakit X dan wabak bermaksud mengambil langkah untuk melindungi diri sendiri sahaja sebelum ianya berlaku	1	1	1	1	1		5	1	1
K10. Sebahagian persediaan isi rumah menghadapi Penyakit X dan wabak ialah menyimpan maklumat untuk mendapatkan bantuan jika Penyakit X atau wabak berlaku	1	1	1	1	1		5	1	1
K11. Sebahagian persediaan isi rumah untuk menghadapi Penyakit X dan wabak ialah merancang tindakan yang perlu diambil jika Penyakit X atau wabak berlaku	1	1	1	1	1		5	1	1
K12. Bekerja dengan haiwan dan produk haiwan (seperti daging, kulit, dan tanduk) boleh meningkatkan risiko Penyakit X dan wabak merebak dari haiwan kepada manusia	1	1	1	1	1		5	1	1

K13. Makan daging mentah atau daging haiwan liar boleh meningkatkan risiko Penyakit X dan wabak merebak dari haiwan kepada manusia	1	1	1	1	1		5	1	1
K14. Kawasan yang sesak mempunyai risiko yang lebih rendah untuk penyebaran Penyakit X dan wabak	1	1	1	1	1		5	1	1
K15. Orang dengan masalah kesihatan kronik (contoh: asma, kencing manis, barah) berisiko lebih tinggi untuk mengalami sakit teruk jika dijangkiti Penyakit X atau wabak	1	1	1	1	1		5	1	1
K16. Kanak-kanak di bawah 5 tahun berisiko lebih tinggi untuk mengalami sakit teruk jika dijangkiti Penyakit X atau wabak	1	1	1	1	1		5	1	1
A1. Saya dan ahli isi rumah saya berisiko dijangkiti Penyakit X dan wabak	1	1	1	1	1		5	1	1
A2. Saya dan ahli isi rumah saya berisiko menyebarkan Penyakit X dan wabak kepada orang lain	1	1	1	1	1		5	1	1
A3. Saya dan ahli isi rumah berisiko untuk meninggal dunia sekiranya dijangkiti Penyakit X dan wabak	1	1	1	1	1		5	1	1
A4. Penyakit X dan wabak berisiko menyebabkan tekanan jiwa atau masalah kesihatan mental kepada saya dan ahli isi rumah saya	1	1	1	1	1		5	1	1
A5. Penyakit X dan wabak tidak akan menjejaskan sumber pendapatan dan mata pencarian saya serta ahli isi rumah saya	1	1	1	1	1		5	1	1
A6. Tindakan persediaan dapat melindungi diri sendiri dan ahli isi rumah daripada jangkitan Penyakit X dan wabak	1	1	1	1	1		5	1	1
A7. Tindakan persediaan menghadapi Penyakit X dan wabak membantu saya dan ahli isi rumah mengetahui apa yang perlu dilakukan jika dijangkiti Penyakit X dan wabak	1	1	1	1	1		5	1	1
A8. Persediaan menghadapi Penyakit X dan wabak adalah tanggungjawab kerajaan sepenuhnya	1	1	1	1	1		5	1	1
A9. Saya dan ahli isi rumah berupaya mengambil langkah untuk bersiap sedia menghadapi Penyakit X dan wabak dengan sendiri	1	1	1	1	1		5	1	1

A10. Saya dan ahli isi rumah berpendapat, buat masa sekarang, persediaan menghadapi Penyakit X dan wabak adalah tidak penting	1	1	1	1	1		5	1	1
A11. Saya dan ahli isi rumah berpendapat, persediaan menghadapi Penyakit X dan wabak memerlukan belanja yang banyak	1	1	1	1	1		5	1	1
A12. Saya dan ahli isi rumah tidak suka mematuhi arahan daripada pihak berkuasa kesihatan seperti kuarantin, pakai pelitup muka, atau penjarakan fizikal, jika dijangkiti Penyakit X atau wabak	1	1	1	1	1		5	1	1
A13. Saya dan ahli isi rumah tidak suka menerima rawatan perubatan seperti diambil darah, makan ubat, atau dicucuk vaksin, jika dijangkiti Penyakit X atau wabak	1	1	1	1	1		5	1	1
A14. Saya dan ahli isi rumah tidak cukup masa untuk mendapatkan rawatan kesihatan jika terkena Penyakit X dan wabak	1	1	1	1	1		5	1	1
A15. Saya dan ahli isi rumah tidak cukup wang untuk mendapatkan rawatan kesihatan jika terkena Penyakit X dan wabak	1	1	1	1	1		5	1	1
A16. Saya dan ahli isi rumah tidak ada pengangkutan untuk mendapatkan rawatan kesihatan jika terkena Penyakit X dan wabak	1	1	1	1	1		5	1	1
P1. Saya dan ahli isi rumah telah menyediakan senarai nombor telefon perkhidmatan kecemasan dan kontak penting	1	1	1	1	1		5	1	1
P2. Saya dan ahli isi rumah telah menyediakan senarai maklumat kesihatan ahli isi rumah	1	1	1	1	1		5	1	1
P3. Saya dan ahli isi rumah telah mengenal pasti siapa untuk dihubungi apabila mengesyaki kemungkinan Penyakit X dan wabak	1	1	1	1	1		5	1	1
P4. Saya dan ahli isi rumah telah mengenal pasti cara untuk kami menerima maklumat berkaitan Penyakit X dan wabak	1	1	1	1	1		5	1	1
P5. Saya dan ahli isi rumah telah menyediakan bekalan makanan dan air cukup untuk sekurang-kurangnya 3 hari	1	1	1	1	1		5	1	1

P6. Saya dan ahli isi rumah telah memilih bilik atau ruang untuk pengasingan ahli rumah yang sakit dijangkiti Penyakit X atau wabak	1	1	1	1	1		5	1	1
P7. Saya dan ahli isi rumah telah menyediakan ubat demam di dalam kit wabak	1	1	1	1	1		5	1	1
P8. Saya dan ahli isi rumah telah menyediakan barangan penjagaan luka di dalam kit wabak	1	1	1	1	1		5	1	1
P9. Saya dan ahli isi rumah saya telah menyediakan barangan pembasmi kuman di dalam kit wabak	1	1	1	1	1		5	1	1
P10. Saya dan ahli isi rumah saya telah menyediakan peralatan perlindungan diri di dalam kit wabak	1	1	1	1	1		5	1	1
P11. Nomborkan mengikut turutan yang betul langkah-langkah cuci tangan menggunakan sabun atau sanitizer	1	1	1	1	1		5	1	1
P12. Nomborkan mengikut turutan yang betul langkah-langkah apabila terdapat ahli keluarga yang sakit tenat disyaki akibat jangkitan Penyakit X atau wabak	1	1	1	1	1		5	1	1
P13. Nomborkan mengikut turutan yang betul langkah-langkah apabila terdapat ahli keluarga yang tiba-tiba rebah dan tidak sedarkan diri	1	1	1	1	1		5	1	1
							**SCVI/Ave**	**1.0**	
**Proportion of clarity**	1	1	1	1	1		**SCVI/UA**		**1.0**
	**Average of proportion of items judged as clear across experts**					**1.0**			

S3.6 Table. Sociodemographic backgrounds of raters in face validation (n=14).

Rater	Age	Ethnic group	Subgroups	Education level	Occupation	Village position and responsibility
1	30	Proto-Malay	Temuan	Primary education	Agricultural worker (private sector)	Village leader
2	27	Proto-Malay	Temuan	Secondary education	Homemaker	Village committee member
3	29	Proto-Malay	Temuan	Secondary education	Homemaker	-
4	38	Proto-Malay	Temuan	Tertiary education	Sales manager	Village leader
5	35	Proto-Malay	Temuan	Secondary education	Homemaker	Village committee member
6	27	Proto-Malay	Temuan	Tertiary education	Decorator	Village committee member
7	26	Senoi	Jahut	Tertiary education	Contract employee (personal MySTEP program)	-
8	36	Senoi	Semai	Secondary education	General support assistant (pembantu khidmat am)	-
9	54	Proto-Malay	Jakun	Secondary education	Security guard	
10	50	Senoi	Semai	Secondary education	Health care assistant (pembantu perawatan kesihatan)	-
11	41	Proto-Malay	Semelai	Tertiary education	Assistant medical officer (penolong pegawai perubatan)	
12	36	Proto-Malay	Semelai	Tertiary education	Nurse	-
13	43	Senoi	Semai	Secondary education	General support assistant (pembantu khidmat am)	Village committee member
14	55	Senoi	Temiar	Primary education	General support assistant (pembantu khidmat am)	-

S3.7 Table. Clarity ratings of the X-SIAGA program materials by Orang Asli community members (n=14).

X-SIAGA materials	Rater															Raters in agreement	I-FVI	UA
	1	2	3	4	5	6	7	8	9	10	11	12	13	14				
1. Lecture: What is Disease X?	1	1	1	1	1	1	1	1	1	1	1	1	1	1		14	1	1
2. Lecture: What is an outbreak?	1	1	1	1	1	1	1	1	1	1	1	1	1	1		14	1	1
3. Lecture: When can Disease X emerge and spread into an outbreak?	1	1	1	1	1	1	1	1	1	1	1	1	1	1		14	1	1
4. Lecture: Where can Disease X and outbreaks spread?	1	1	1	1	1	1	1	1	1	1	1	1	1	1		14	1	1
5. Lecture: How can Disease X and outbreaks spread?	1	1	1	1	1	1	1	1	1	1	1	1	1	1		14	1	1
6. Video session: How can Disease X and outbreaks spread?	1	1	1	1	1	1	1	1	1	1	1	1	1	1		14	1	1
7. Lecture: Who is at higher risk of Disease X and outbreaks?	1	1	1	1	1	1	1	1	1	1	1	1	1	1		14	1	1
8. Lecture: Why should we be concerned about Disease X?	1	1	1	1	1	1	1	1	1	1	1	1	1	1		14	1	1
9. Lecture: What is meant by "household preparedness for Disease X and outbreaks"?	1	1	1	1	1	1	1	1	1	1	1	1	1	1		14	1	1
10. Lecture: What is the importance of household preparedness for Disease X and outbreaks?	1	1	1	1	1	1	1	1	1	1	1	1	1	1		14	1	1
11. Lecture: Why are Orang Asli communities at risk of Disease X and outbreaks?	1	1	1	1	1	1	1	1	1	1	1	1	1	1		14	1	1
12. Lecture: Who is responsible for taking preparedness actions?	1	1	1	1	1	1	1	1	1	1	1	1	1	1		14	1	1
13. Lecture: How can households prepare for Disease X and outbreaks?	1	1	1	1	1	1	1	1	1	1	1	1	1	1		14	1	1
14. Video session: Understanding disease and outbreak spread and household preparedness (Nipah Virus Outbreak)	1	1	1	1	1	1	1	1	1	1	1	1	1	1		14	1	1

15. Video session: Understanding disease and outbreak spread and household preparedness (Ebola Virus Outbreak)	1	1	1	1	1	1	1	1	1	1	1	1	1	1		14	1	1
16. Video session: Understanding disease and outbreak spread and household preparedness (COVID-19 Pandemic)	1	1	1	1	1	1	1	1	1	1	1	1	1	1		14	1	1
17. Hands-on session: 7 steps of proper handwashing	1	1	1	1	1	1	1	1	1	1	1	1	1	1		14	1	1
18. Hands-on session: Developing household planning for Disease X and outbreaks using the template provided in the booklet	1	1	1	1	1	1	1	1	1	1	1	1	1	1		14	1	1
19. Hands-on session: Learning symptoms of potential Disease X and signs of outbreaks through puzzle activities	1	0	1	1	1	1	1	1	1	0	1	1	1	1		12	0.85	0
20. Hands-on session: Learning steps for handling suspected sick or dead animals and family members during Disease X or outbreaks through puzzle activities	1	0	1	1	1	1	1	1	1	0	1	1	1	1		12	0.85	0
21. Puzzle explanation: Handling sick or dead animals suspected of Disease X or outbreaks	1	1	1	1	1	1	1	1	1	1	1	1	1	1		14	1	1
22. Puzzle explanation: Handling family members suspected of having Disease X or outbreaks	1	0	1	1	1	1	1	1	1	0	1	1	1	1		12	0.85	0
23. Lecture: Cleaning and disinfecting the home and refilling the outbreak kit after the outbreak	1	1	1	1	1	1	1	1	1	1	1	1	1	1		14	1	1
24. Lecture: Hands-only CPR	1	0	1	1	1	1	1	1	1	0	1	1	1	1		12	0.85	0
25. Hands-on session: Hands-only CPR	1	0	1	1	1	1	1	1	1	0	1	1	1	1		12	0.85	0
26. Simulation exercise 1 (handling sick or dead animals during a suspected Disease X or outbreak)	1	1	1	1	1	1	1	1	1	1	1	1	1	1		14	1	1

27. Simulation exercise 2 (handling sick family members during a suspected Disease X or outbreak)	1	1	1	1	1	1	1	1	1	1	1	1	1	1		14	1	1
28. Simulation exercise 3 (performing hands-only CPR for life-saving measures)	1	1	1	1	1	1	1	1	1	1	1	1	1	1		14	1	1
29. Game-based learning: Wabak X instructional leaflet	1	1	1	1	1	0	1	0	1	1	1	1	1	1		12	0.85	0
30. Game-based learning: Wabak X game mechanics	1	1	1	1	1	1	1	1	1	1	1	1	1	1		14	1	1
31. Game-based learning: Lessons from Wabak X gameplay	1	1	1	1	1	1	1	1	1	1	1	1	1	1		14	1	1
32. Game-based learning: Wabak X illustrations	1	1	1	1	1	1	1	1	1	1	1	1	1	1		14	1	1
																**SFVI/Ave**	**0.97**	
**Proportion of clarity**	1	0.84	1	1	1	0.96	1	0.96	1	0.84	1	1	1	1		**SFVI/UA**		**0.81**
	**Average of proportion of contents judged as clear across raters**														**0.97**			

S3.8 Table. Comprehensibility ratings of the X-SIAGA materials by Orang Asli community members (n = 14).

X-SIAGA materials	Rater															Raters in agreement	I-FVI	UA
	1	2	3	4	5	6	7	8	9	10	11	12	13	14				
1. Lecture: What is Disease X?	1	1	1	1	1	1	1	1	1	1	1	1	1	1		14	1	1
2. Lecture: What is an outbreak?	1	1	1	1	1	1	1	1	1	1	1	1	1	1		14	1	1
3. Lecture: When can Disease X emerge and spread into an outbreak?	1	1	1	1	1	1	1	1	1	1	1	1	1	1		14	1	1
4. Lecture: Where can Disease X and outbreaks spread?	1	1	1	1	1	1	1	1	1	1	1	1	1	1		14	1	1
5. Lecture: How can Disease X and outbreaks spread?	1	1	1	1	1	1	1	1	1	1	1	1	1	1		14	1	1
6. Video session: How can Disease X and outbreaks spread?	1	1	1	1	1	1	1	1	1	1	1	1	1	1		14	1	1
7. Lecture: Who is at higher risk of Disease X and outbreaks?	1	1	1	1	1	1	1	1	1	1	1	1	1	1		14	1	1
8. Lecture: Why should we be concerned about Disease X?	1	1	1	1	1	1	1	1	1	1	1	1	1	1		14	1	1
9. Lecture: What is meant by "household preparedness for Disease X and outbreaks"?	1	1	1	1	1	1	1	1	1	1	1	1	1	1		14	1	1
10. Lecture: What is the importance of household preparedness for Disease X and outbreaks?	1	1	1	1	1	1	1	1	1	1	1	1	1	1		14	1	1
11. Lecture: Why are Orang Asli communities at risk of Disease X and outbreaks?	1	1	1	1	1	1	1	1	1	1	1	1	1	1		14	1	1
12. Lecture: Who is responsible for taking preparedness actions?	1	1	1	1	1	1	1	1	1	1	1	1	1	1		14	1	1
13. Lecture: How can households prepare for Disease X and outbreaks?	1	1	1	1	1	1	1	1	1	1	1	1	1	1		14	1	1
14. Video session: Understanding disease and outbreak spread and household preparedness (Nipah Virus Outbreak)	1	1	1	1	1	1	1	1	1	1	1	1	1	1		14	1	1

15. Video session: Understanding disease and outbreak spread and household preparedness (Ebola Virus Outbreak)	1	1	1	1	1	1	1	1	1	1	1	1	1	1		14	1	1
16. Video session: Understanding disease and outbreak spread and household preparedness (COVID-19 Pandemic)	1	1	1	1	1	1	1	1	1	1	1	1	1	1		14	1	1
17. Hands-on session: 7 steps of proper handwashing	1	1	1	1	1	1	1	1	1	1	1	1	1	1		14	1	1
18. Hands-on session: Developing household planning for Disease X and outbreaks using the template provided in the booklet	1	1	1	1	1	1	1	1	1	1	1	1	1	1		14	1	1
19. Hands-on session: Learning symptoms of potential Disease X and signs of outbreaks through puzzle activities	1	1	1	1	1	1	1	1	1	1	1	1	1	1		14	1	1
20. Hands-on session: Learning steps for handling suspected sick or dead animals and family members during Disease X or outbreaks through puzzle activities	1	0	1	1	1	1	1	1	1	0	1	1	1	1		12	0.85	0
21. Puzzle explanation: Handling sick or dead animals suspected of Disease X or outbreaks	1	1	0	1	1	1	1	1	1	1	1	1	1	1		13	0.92	0
22. Puzzle explanation: Handling family members suspected of having Disease X or outbreaks	1	1	0	1	1	1	1	1	1	0	1	1	1	1		12	0.85	0
23. Lecture: Cleaning and disinfecting the home and refilling the outbreak kit after the outbreak	1	1	1	1	1	1	1	1	1	1	1	1	1	1		14	1	1
24. Lecture: Hands-only CPR	1	0	0	1	1	1	1	1	1	1	1	1	1	1		12	0.85	0
25. Hands-on session: Hands-only CPR	1	0	0	1	1	1	1	1	1	1	1	1	1	1		12	0.85	0
26. Simulation exercise 1 (handling sick or dead animals during a suspected Disease X or outbreak)	1	1	1	1	1	1	1	1	1	1	1	1	1	1		14	1	1

27. Simulation exercise 2 (handling sick family members during a suspected Disease X or outbreak)	1	1	1	1	1	1	1	1	1	1	1	1	1	1		14	1	1
28. Simulation exercise 3 (performing hands-only CPR for life-saving measures)	1	1	1	1	1	1	1	1	1	1	1	1	1	1		14	1	1
29. Game-based learning: Wabak X instructional leaflet	1	1	1	1	1	0	1	0	1	1	1	1	1	1		12	0.85	0
30. Game-based learning: Wabak X game mechanics	1	1	1	1	1	1	1	1	1	1	1	1	1	1		14	1	1
31. Game-based learning: Lessons from Wabak X gameplay	1	1	1	1	1	1	1	1	1	1	1	1	1	1		14	1	1
32. Game-based learning: Wabak X illustrations	1	1	1	1	1	1	1	1	1	1	1	1	1	1		14	1	1
																**SFVI/Ave**	**0.97**	
**Proportion of comprehensibility**	1	0.90	0.87	1	1	0.96	1	0.96	1	0.93	1	1	1	1		**SFVI/UA**		**0.81**
	**Average of proportion of contents judged as comprehensible across raters**														**0.97**			

S3.9 Table. Relevance to community needs ratings of the X-SIAGA materials by Orang Asli community members (n = 14).

X-SIAGA materials	Rater															Raters in agreement	I-FVI	UA
	1	2	3	4	5	6	7	8	9	10	11	12	13	14				
1. Lecture: What is Disease X?	1	1	1	1	1	1	1	1	1	1	1	1	1	1		14	1	1
2. Lecture: What is an outbreak?	1	1	1	1	1	1	1	1	1	1	1	1	1	1		14	1	1
3. Lecture: When can Disease X emerge and spread into an outbreak?	1	1	1	1	1	1	1	1	1	1	1	1	1	1		14	1	1
4. Lecture: Where can Disease X and outbreaks spread?	1	1	1	1	1	1	1	1	1	1	1	1	1	1		14	1	1
5. Lecture: How can Disease X and outbreaks spread?	1	1	1	1	1	1	1	1	1	1	1	1	1	1		14	1	1
6. Video session: How can Disease X and outbreaks spread?	1	1	1	1	1	1	1	1	1	1	1	1	1	1		14	1	1
7. Lecture: Who is at higher risk of Disease X and outbreaks?	1	1	1	1	1	1	1	1	1	1	1	1	1	1		14	1	1
8. Lecture: Why should we be concerned about Disease X?	1	1	1	1	1	1	1	1	1	1	1	1	1	1		14	1	1
9. Lecture: What is meant by "household preparedness for Disease X and outbreaks"?	1	1	1	1	1	1	1	1	1	1	1	1	1	1		14	1	1
10. Lecture: What is the importance of household preparedness for Disease X and outbreaks?	1	1	1	1	1	1	1	1	1	1	1	1	1	1		14	1	1
11. Lecture: Why are Orang Asli communities at risk of Disease X and outbreaks?	1	1	1	1	1	1	1	1	1	1	1	1	1	1		14	1	1
12. Lecture: Who is responsible for taking preparedness actions?	1	1	1	1	1	1	1	1	1	1	1	1	1	1		14	1	1
13. Lecture: How can households prepare for Disease X and outbreaks?	1	1	1	1	1	1	1	1	1	1	1	1	1	1		14	1	1
14. Video session: Understanding disease and outbreak spread and household preparedness (Nipah Virus Outbreak)	1	1	1	1	1	1	1	1	1	1	1	1	1	1		14	1	1

15. Video session: Understanding disease and outbreak spread and household preparedness (Ebola Virus Outbreak)	1	1	1	1	1	1	1	1	1	1	1	1	1	1		14	1	1
16. Video session: Understanding disease and outbreak spread and household preparedness (COVID-19 Pandemic)	1	1	1	1	1	1	1	1	1	1	1	1	1	1		14	1	1
17. Hands-on session: 7 steps of proper handwashing	1	1	1	1	1	1	1	1	1	1	1	1	1	1		14	1	1
18. Hands-on session: Developing household planning for Disease X and outbreaks using the template provided in the booklet	1	1	1	1	1	1	1	1	1	1	1	1	1	1		14	1	1
19. Hands-on session: Learning symptoms of potential Disease X and signs of outbreaks through puzzle activities	1	1	1	1	1	1	1	1	1	1	1	1	1	1		14	1	1
20. Hands-on session: Learning steps for handling suspected sick or dead animals and family members during Disease X or outbreaks through puzzle activities	1	1	1	1	1	1	1	1	1	1	1	1	1	1		14	1	1
21. Puzzle explanation: Handling sick or dead animals suspected of Disease X or outbreaks	1	1	1	1	1	1	1	1	1	1	1	1	1	1		14	1	1
22. Puzzle explanation: Handling family members suspected of having Disease X or outbreaks	1	1	1	1	1	1	1	1	1	1	1	1	1	1		14	1	1
23. Lecture: Cleaning and disinfecting the home and refilling the outbreak kit after the outbreak	1	1	1	1	1	1	1	1	1	1	1	1	1	1		14	1	1
24. Lecture: Hands-only CPR	1	1	1	1	1	1	1	1	1	1	1	1	1	1		14	1	1
25. Hands-on session: Hands-only CPR	1	1	1	1	1	1	1	1	1	1	1	1	1	1		14	1	1
26. Simulation exercise 1 (handling sick or dead animals during a suspected Disease X or outbreak)	1	1	1	1	1	1	1	1	1	1	1	1	1	1		14	1	1

27. Simulation exercise 2 (handling sick family members during a suspected Disease X or outbreak)	1	1	1	1	1	1	1	1	1	1	1	1	1	1		14	1	1
28. Simulation exercise 3 (performing hands-only CPR for life-saving measures)	1	1	1	1	1	1	1	1	1	1	1	1	1	1		14	1	1
29. Game-based learning: Wabak X instructional leaflet	1	1	1	1	1	1	1	1	1	1	1	1	1	1		14	1	1
30. Game-based learning: Wabak X game mechanics	1	1	1	1	1	1	1	0	1	1	1	1	1	1		13	0.92	0
31. Game-based learning: Lessons from Wabak X gameplay	1	1	1	1	1	1	1	1	1	1	1	1	1	1		14	1	1
32. Game-based learning: Wabak X illustrations	1	1	1	1	1	1	1	1	1	1	1	1	1	1		14	1	1
																**SFVI/Ave**	**0.99**	
**Proportion of relevance to community needs**	1	1	1	1	1	1	1	0.96	1	1	1	1	1	1		**SFVI/UA**		**0.93**
	**Average of proportion of contents judged as relevant to community needs across raters**														**0.99**			

S3.10 Table. Clarity ratings of the HOPE items by Orang Asli community members (n = 14).

HOPE items	Rater															Raters in agreement	I-FVI	UA
	1	2	3	4	5	6	7	8	9	10	11	12	13	14				
K1. Penyakit X adalah penyakit berjangkit yang baharu dan belum dikenalpasti	1	1	1	1	1	1	1	1	1	1	1	1	1	1		14	1	1
K2. Penyakit X adalah penyakit yang boleh terjadi di mana-mana sahaja di dunia	1	1	1	1	1	1	1	1	1	1	1	1	1	1		14	1	1
K3. Haiwan tidak mungkin menyebarkan Penyakit X kepada manusia	1	1	1	1	1	1	1	1	1	1	1	1	1	1		14	1	1
K4. Sawan adalah antara gejala jika kuman menyerang paru-paru dan sistem pernafasan	1	1	1	1	1	0	1	0	1	1	1	1	1	1		12	0.85	0
K5. Wabak disyaki berlaku apabila terdapat 2 atau lebih orang yang mengalami gejala sama, dalam tempoh masa yang hampir sama, dan mereka ada kaitan dengan tempat atau orang yang sama	1	1	1	1	1	1	1	0	1	1	1	1	1	1		13	0.92	0
K6. Wabak Penyakit X boleh diramalkan bila akan berlaku	1	1	1	1	1	1	1	1	1	1	1	1	1	1		14	1	1
K7. Wabak Penyakit X diramalkan lebih teruk dan merbahaya daripada COVID-19	1	1	1	1	1	1	1	1	1	1	1	1	1	1		14	1	1
K8. Hanya pihak berkuasa kesihatan sahaja yang boleh melaporkan wabak	1	1	1	1	1	1	1	1	1	1	1	1	1	1		14	1	1
K9. Persediaan isi rumah dalam menghadapi Penyakit X dan wabak bermaksud mengambil langkah untuk melindungi diri sendiri sahaja sebelum ianya berlaku	1	1	1	1	1	0	1	0	1	1	1	1	1	1		12	0.85	0
K10. Sebahagian persediaan isi rumah menghadapi Penyakit X dan wabak ialah menyimpan maklumat untuk	1	1	1	1	1	0	1	0	1	1	1	1	1	1		12	0.85	0

mendapatkan bantuan jika Penyakit X atau wabak berlaku																		
K11. Sebahagian persediaan isi rumah untuk menghadapi Penyakit X dan wabak ialah merancang tindakan yang perlu diambil jika Penyakit X atau wabak berlaku	1	1	1	1	1	0	1	0	1	1	1	1	1	1		12	0.85	0
K12. Bekerja dengan haiwan dan produk haiwan (seperti daging, kulit, dan tanduk) boleh meningkatkan risiko Penyakit X dan wabak merebak dari haiwan kepada manusia	1	1	1	1	1	0	1	0	1	1	1	1	1	1		12	0.85	0
K13. Makan daging mentah atau daging haiwan liar boleh meningkatkan risiko Penyakit X dan wabak merebak dari haiwan kepada manusia	1	1	1	1	1	1	1	1	1	1	1	1	1	1		14	1	1
K14. Kawasan yang sesak mempunyai risiko yang lebih rendah untuk penyebaran Penyakit X dan wabak	1	1	1	1	1	1	1	1	1	1	1	1	1	1		14	1	1
K15. Orang dengan masalah kesihatan kronik (contoh: asma, kencing manis, barah) berisiko lebih tinggi untuk mengalami sakit teruk jika dijangkiti Penyakit X atau wabak	1	1	1	1	1	1	1	1	1	1	1	1	1	1		14	1	1
K16. Kanak-kanak di bawah 5 tahun berisiko lebih tinggi untuk mengalami sakit teruk jika dijangkiti Penyakit X atau wabak	1	1	1	1	1	1	1	1	1	1	1	1	1	1		14	1	1
A1. Saya dan ahli isi rumah saya berisiko dijangkiti Penyakit X dan wabak	1	1	1	1	1	1	1	1	1	1	1	1	1	1		14	1	1
A2. Saya dan ahli isi rumah saya berisiko menyebarkan Penyakit X dan wabak kepada orang lain	1	1	1	1	1	1	1	1	1	1	1	1	1	1		14	1	1

A3. Saya dan ahli isi rumah berisiko untuk meninggal dunia sekiranya dijangkiti Penyakit X dan wabak	1	1	1	1	1	1	1	1	1	1	1	1	1	1		14	1	1
A4. Penyakit X dan wabak berisiko menyebabkan tekanan jiwa atau masalah kesihatan mental kepada saya dan ahli isi rumah saya	1	1	1	1	1	1	1	1	1	1	1	1	1	1		14	1	1
A5. Penyakit X dan wabak tidak akan menjejaskan sumber pendapatan dan mata pencarian saya serta ahli isi rumah saya	1	1	1	1	1	1	1	1	1	1	1	1	1	1		14	1	1
A6. Tindakan persediaan dapat melindungi diri sendiri dan ahli isi rumah daripada jangkitan Penyakit X dan wabak	1	1	1	1	1	1	1	1	1	1	1	1	1	1		14	1	1
A7. Tindakan persediaan menghadapi Penyakit X dan wabak membantu saya dan ahli isi rumah mengetahui apa yang perlu dilakukan jika dijangkiti Penyakit X dan wabak	1	1	1	1	1	1	1	1	1	1	1	1	1	1		14	1	1
A8. Persediaan menghadapi Penyakit X dan wabak adalah tanggungjawab kerajaan sepenuhnya	1	1	1	1	1	1	1	1	1	1	1	1	1	1		14	1	1
A9. Saya dan ahli isi rumah berupaya mengambil langkah untuk bersiap sedia menghadapi Penyakit X dan wabak dengan sendiri	1	1	1	1	1	1	1	1	1	1	1	1	1	1		14	1	1
A10. Saya dan ahli isi rumah berpendapat, buat masa sekarang, persediaan menghadapi Penyakit X dan wabak adalah tidak penting	1	1	1	1	1	1	1	1	1	1	1	1	1	1		14	1	1
A11. Saya dan ahli isi rumah berpendapat, persediaan menghadapi Penyakit X dan wabak memerlukan belanja yang banyak	1	1	1	1	1	1	1	1	1	1	1	1	1	1		14	1	1
A12. Saya dan ahli isi rumah tidak suka mematuhi arahan daripada pihak berkuasa kesihatan seperti kuarantin, pakai pelitup	1	1	1	1	1	1	1	1	1	1	1	1	1	1		14	1	1

muka, atau penjarakan fizikal, jika dijangkiti Penyakit X atau wabak																		
A13. Saya dan ahli isi rumah tidak suka menerima rawatan perubatan seperti diambil darah, makan ubat, atau dicucuk vaksin, jika dijangkiti Penyakit X atau wabak	1	1	1	1	1	1	1	1	1	1	1	1	1	1		14	1	1
A14. Saya dan ahli isi rumah tidak cukup masa untuk mendapatkan rawatan kesihatan jika terkena Penyakit X dan wabak	1	1	1	1	1	1	1	1	1	1	1	1	1	1		14	1	1
A15. Saya dan ahli isi rumah tidak cukup wang untuk mendapatkan rawatan kesihatan jika terkena Penyakit X dan wabak	1	1	1	1	1	1	1	1	1	1	1	1	1	1		14	1	1
A16. Saya dan ahli isi rumah tidak ada pengangkutan untuk mendapatkan rawatan kesihatan jika terkena Penyakit X dan wabak	1	1	1	1	1	1	1	1	1	1	1	1	1	1		14	1	1
P1. Saya dan ahli isi rumah telah menyediakan senarai nombor telefon perkhidmatan kecemasan dan kontak penting	1	1	1	1	1	1	1	1	1	1	1	1	1	1		14	1	1
P2. Saya dan ahli isi rumah telah menyediakan senarai maklumat kesihatan ahli isi rumah	1	1	1	1	1	1	1	1	1	1	1	1	1	1		14	1	1
P3. Saya dan ahli isi rumah telah mengenal pasti siapa untuk dihubungi apabila mengesyaki kemungkinan Penyakit X dan wabak	1	1	1	1	1	1	1	1	1	1	1	1	1	1		14	1	1
P4. Saya dan ahli isi rumah telah mengenal pasti cara untuk kami menerima maklumat berkaitan Penyakit X dan wabak	1	1	1	1	1	1	1	0	1	1	1	1	1	1		13	0.92	0

P5. Saya dan ahli isi rumah telah menyediakan bekalan makanan dan air cukup untuk sekurang-kurangnya 3 hari	1	1	1	1	1	1	1	0	1	1	1	1	1	1		13	0.92	0
P6. Saya dan ahli isi rumah telah memilih bilik atau ruang untuk pengasingan ahli rumah yang sakit dijangkiti Penyakit X atau wabak	1	1	1	1	1	1	1	0	1	1	1	1	1	1		13	0.92	0
P7. Saya dan ahli isi rumah telah menyediakan ubat demam di dalam kit wabak	1	1	1	1	1	1	1	0	1	1	1	1	1	1		13	0.92	0
P8. Saya dan ahli isi rumah telah menyediakan barangan penjagaan luka di dalam kit wabak	1	1	1	1	1	1	1	1	1	1	1	1	1	1		14	1	1
P9. Saya dan ahli isi rumah saya telah menyediakan barangan pembasmi kuman di dalam kit wabak	1	1	1	1	1	1	1	1	1	1	1	1	1	1		14	1	1
P10. Saya dan ahli isi rumah saya telah menyediakan peralatan perlindungan diri di dalam kit wabak	1	1	1	1	1	1	1	1	1	1	1	1	1	1		14	1	1
P11. Nomborkan mengikut turutan yang betul langkah-langkah cuci tangan menggunakan sabun atau sanitizer	1	1	1	1	1	1	1	1	1	1	1	1	1	1		14	1	1
P12. Nomborkan mengikut turutan yang betul langkah-langkah apabila terdapat ahli keluarga yang sakit tenat disyaki akibat jangkitan Penyakit X atau wabak	1	1	1	1	1	1	1	1	1	1	1	1	1	1		14	1	1
P13. Nomborkan mengikut turutan yang betul langkah-langkah apabila terdapat ahli keluarga yang tiba-tiba rebah dan tidak sedarkan diri	1	1	1	1	1	1	1	1	1	1	1	1	1	1		14	1	1
																**SFVI/Ave**	**0.98**	
**Proportion of clarity**	1	1	1	1	1	0.89	1	0.79	1	1	1	1	1	1		**SFVI/UA**		**0.79**
	**Average of proportion of items judged as clear across raters**														**0.97**			

S3.11 Table. Comprehensibility ratings of the HOPE items by Orang Asli community members (n = 14).

HOPE items	Rater															Raters in agreement	I-FVI	UA
	1	2	3	4	5	6	7	8	9	10	11	12	13	14				
K1. Penyakit X adalah penyakit berjangkit yang baharu dan belum dikenalpasti	1	1	1	1	1	1	1	1	1	1	1	1	1	1		14	1	1
K2. Penyakit X adalah penyakit yang boleh terjadi di mana-mana sahaja di dunia	1	1	1	1	1	1	1	1	1	1	1	1	1	1		14	1	1
K3. Haiwan tidak mungkin menyebarkan Penyakit X kepada manusia	1	1	1	1	1	1	1	1	1	1	1	1	1	1		14	1	1
K4. Sawan adalah antara gejala jika kuman menyerang paru-paru dan sistem pernafasan	1	1	1	1	1	1	1	0	1	1	1	1	1	1		13	0.92	0
K5. Wabak disyaki berlaku apabila terdapat 2 atau lebih orang yang mengalami gejala sama, dalam tempoh masa yang hampir sama, dan mereka ada kaitan dengan tempat atau orang yang sama	1	1	1	1	1	1	1	0	1	1	1	1	1	1		13	0.92	0
K6. Wabak Penyakit X boleh diramalkan bila akan berlaku	1	1	1	1	1	1	1	1	1	1	1	1	1	1		14	1	1
K7. Wabak Penyakit X diramalkan lebih teruk dan merbahaya daripada COVID-19	1	1	1	1	1	1	1	1	1	1	1	1	1	1		14	1	1
K8. Hanya pihak berkuasa kesihatan sahaja yang boleh melaporkan wabak	1	1	1	1	1	1	1	1	1	1	1	1	1	1		14	1	1
K9. Persediaan isi rumah dalam menghadapi Penyakit X dan wabak bermaksud mengambil langkah untuk melindungi diri sendiri sahaja sebelum ianya berlaku	1	1	1	1	1	1	1	0	1	1	1	1	1	1		13	0.92	0
K10. Sebahagian persediaan isi rumah menghadapi Penyakit X dan wabak ialah menyimpan maklumat untuk	1	1	1	1	1	1	1	0	1	1	1	1	1	1		13	0.92	0

mendapatkan bantuan jika Penyakit X atau wabak berlaku																	
K11. Sebahagian persediaan isi rumah untuk menghadapi Penyakit X dan wabak ialah merancang tindakan yang perlu diambil jika Penyakit X atau wabak berlaku	1	1	1	1	1	1	1	0	1	1	1	1	1	1		13	0.92	0
K12. Bekerja dengan haiwan dan produk haiwan (seperti daging, kulit, dan tanduk) boleh meningkatkan risiko Penyakit X dan wabak merebak dari haiwan kepada manusia	1	1	1	1	1	1	1	0	1	1	1	1	1	1		13	0.92	0
K13. Makan daging mentah atau daging haiwan liar boleh meningkatkan risiko Penyakit X dan wabak merebak dari haiwan kepada manusia	1	1	1	1	1	1	1	1	1	1	1	1	1	1		14	1	1
K14. Kawasan yang sesak mempunyai risiko yang lebih rendah untuk penyebaran Penyakit X dan wabak	1	1	1	1	1	1	1	1	1	1	1	1	1	1		14	1	1
K15. Orang dengan masalah kesihatan kronik (contoh: asma, kencing manis, barah) berisiko lebih tinggi untuk mengalami sakit teruk jika dijangkiti Penyakit X atau wabak	1	1	1	1	1	1	1	1	1	1	1	1	1	1		14	1	1
K16. Kanak-kanak di bawah 5 tahun berisiko lebih tinggi untuk mengalami sakit teruk jika dijangkiti Penyakit X atau wabak	1	1	1	1	1	1	1	1	1	1	1	1	1	1		14	1	1
A1. Saya dan ahli isi rumah saya berisiko dijangkiti Penyakit X dan wabak	1	1	1	1	1	1	1	1	1	1	1	1	1	1		14	1	1
A2. Saya dan ahli isi rumah saya berisiko menyebarkan Penyakit X dan wabak kepada orang lain	1	1	1	1	1	1	1	1	1	1	1	1	1	1		14	1	1

A3. Saya dan ahli isi rumah berisiko untuk meninggal dunia sekiranya dijangkiti Penyakit X dan wabak	1	1	1	1	1	1	1	1	1	1	1	1	1	1		14	1	1
A4. Penyakit X dan wabak berisiko menyebabkan tekanan jiwa atau masalah kesihatan mental kepada saya dan ahli isi rumah saya	1	1	1	1	1	1	1	1	1	1	1	1	1	1		14	1	1
A5. Penyakit X dan wabak tidak akan menjejaskan sumber pendapatan dan mata pencarian saya serta ahli isi rumah saya	1	1	1	1	1	1	1	1	1	1	1	1	1	1		14	1	1
A6. Tindakan persediaan dapat melindungi diri sendiri dan ahli isi rumah daripada jangkitan Penyakit X dan wabak	1	1	1	1	1	1	1	1	1	1	1	1	1	1		14	1	1
A7. Tindakan persediaan menghadapi Penyakit X dan wabak membantu saya dan ahli isi rumah mengetahui apa yang perlu dilakukan jika dijangkiti Penyakit X dan wabak	1	1	1	1	1	1	1	1	1	1	1	1	1	1		14	1	1
A8. Persediaan menghadapi Penyakit X dan wabak adalah tanggungjawab kerajaan sepenuhnya	1	1	1	1	1	1	1	1	1	1	1	1	1	1		14	1	1
A9. Saya dan ahli isi rumah berupaya mengambil langkah untuk bersiap sedia menghadapi Penyakit X dan wabak dengan sendiri	1	1	1	1	1	1	1	1	1	1	1	1	1	1		14	1	1
A10. Saya dan ahli isi rumah berpendapat, buat masa sekarang, persediaan menghadapi Penyakit X dan wabak adalah tidak penting	1	1	1	1	1	1	1	1	1	1	1	1	1	1		14	1	1
A11. Saya dan ahli isi rumah berpendapat, persediaan menghadapi Penyakit X dan wabak memerlukan belanja yang banyak	1	1	1	1	1	1	1	1	1	1	1	1	1	1		14	1	1
A12. Saya dan ahli isi rumah tidak suka mematuhi arahan daripada pihak berkuasa kesihatan seperti kuarantin, pakai pelitup	1	1	1	1	1	1	1	1	1	1	1	1	1	1		14	1	1

muka, atau penjarakan fizikal, jika dijangkiti Penyakit X atau wabak																		
A13. Saya dan ahli isi rumah tidak suka menerima rawatan perubatan seperti diambil darah, makan ubat, atau dicucuk vaksin, jika dijangkiti Penyakit X atau wabak	1	1	1	1	1	1	1	1	1	1	1	1	1	1		14	1	1
A14. Saya dan ahli isi rumah tidak cukup masa untuk mendapatkan rawatan kesihatan jika terkena Penyakit X dan wabak	1	1	1	1	1	1	1	1	1	1	1	1	1	1		14	1	1
A15. Saya dan ahli isi rumah tidak cukup wang untuk mendapatkan rawatan kesihatan jika terkena Penyakit X dan wabak	1	1	1	1	1	1	1	1	1	1	1	1	1	1		14	1	1
A16. Saya dan ahli isi rumah tidak ada pengangkutan untuk mendapatkan rawatan kesihatan jika terkena Penyakit X dan wabak	1	1	1	1	1	1	1	1	1	1	1	1	1	1		14	1	1
P1. Saya dan ahli isi rumah telah menyediakan senarai nombor telefon perkhidmatan kecemasan dan kontak penting	1	1	1	1	1	1	1	1	1	1	1	1	1	1		14	1	1
P2. Saya dan ahli isi rumah telah menyediakan senarai maklumat kesihatan ahli isi rumah	1	1	1	1	1	1	1	1	1	1	1	1	1	1		14	1	1
P3. Saya dan ahli isi rumah telah mengenal pasti siapa untuk dihubungi apabila mengesyaki kemungkinan Penyakit X dan wabak	1	1	1	1	1	1	1	1	1	1	1	1	1	1		14	1	1
P4. Saya dan ahli isi rumah telah mengenal pasti cara untuk kami menerima maklumat berkaitan Penyakit X dan wabak	1	1	1	1	1	1	1	0	1	1	1	1	1	1		13	0.92	0

P5. Saya dan ahli isi rumah telah menyediakan bekalan makanan dan air cukup untuk sekurang-kurangnya 3 hari	1	1	1	1	1	1	1	0	1	1	1	1	1	1		13	0.92	0
P6. Saya dan ahli isi rumah telah memilih bilik atau ruang untuk pengasingan ahli rumah yang sakit dijangkiti Penyakit X atau wabak	1	1	1	1	1	1	1	0	1	1	1	1	1	1		13	0.92	0
P7. Saya dan ahli isi rumah telah menyediakan ubat demam di dalam kit wabak	1	1	1	1	1	1	1	0	1	1	1	1	1	1		13	0.92	0
P8. Saya dan ahli isi rumah telah menyediakan barangan penjagaan luka di dalam kit wabak	1	1	1	1	1	1	1	1	1	1	1	1	1	1		14	1	1
P9. Saya dan ahli isi rumah saya telah menyediakan barangan pembasmi kuman di dalam kit wabak	1	1	1	1	1	1	1	1	1	1	1	1	1	1		14	1	1
P10. Saya dan ahli isi rumah saya telah menyediakan peralatan perlindungan diri di dalam kit wabak	1	1	1	1	1	1	1	1	1	1	1	1	1	1		14	1	1
P11. Nomborkan mengikut turutan yang betul langkah-langkah cuci tangan menggunakan sabun atau sanitizer	1	1	1	1	1	1	1	1	1	1	1	1	1	1		14	1	1
P12. Nomborkan mengikut turutan yang betul langkah-langkah apabila terdapat ahli keluarga yang sakit tenat disyaki akibat jangkitan Penyakit X atau wabak	1	1	1	1	1	1	1	1	1	1	1	1	1	1		14	1	1
P13. Nomborkan mengikut turutan yang betul langkah-langkah apabila terdapat ahli keluarga yang tiba-tiba rebah dan tidak sedarkan diri	1	1	1	1	1	1	1	1	1	1	1	1	1	1		14	1	1
																**SFVI/Ave**	**0.98**	
**Proportion of comprehensibility**	1	1	1	1	1	1	1	0.77	1	1	1	1	1	1		**SFVI/UA**		**0.77**
	**Average of proportion of items judged as comprehensible across raters**														**0.98**			
