# Supplementary material for: Development of X-SIAGA: A disease X and outbreak preparedness intervention for indigenous households in Selangor, Malaysia
Source: PLoS One. 2026 Mar 30;21(3):e0345785. doi: 10.1371/journal.pone.0345785 (PMC13035154; doi:10.1371/journal.pone.0345785)
Supplement: S4 File — Acceptability and preliminary effectiveness of the X-SIAGA intervention. (PDF) [file pone.0345785.s004.pdf]

S4 File. X-SIAGA pilot testing results.

S4.1 Table. Sociodemographic characteristics of participating households (N=18).

Variable	Min	Max	Median	Mean	SD
Years Living in Village	2	40	22.5	23.1	9.8
Total Household Members	4	11	6	6.2	1.9
Household Members Aged 14 and under	0	6	2	2.2	1.2
Household Members Aged 15 to 64	1	9	3.5	3.9	2.2
Household Members Aged 65 and over	0	2	0	0.3	0.7
Pregnant Household Members	0	1	0	0.2	0.4
Household Members with Chronic Illnesses	0	2	0	0.4	0.7
Disabled Household Members	0	1	0	0.1	0.2
Average Monthly Household Income (MYR)	400	3000	1500	1416.7	609

Note: Min, minimum; Max, maximum; SD, standard deviation; MYR, Malaysian Ringgit.

S4.2 Table. Acceptability of the finalised X-SIAGA intervention (N=18).

Domain	Item	*Percentage Agreement (%)
Affective attitude	Adakah anda suka program X-SIAGA? (1= Sangat tidak suka, 5=Sangat suka)	100.0
	Adakah anda ingin menyertai program X-SIAGA lagi? (1=Tidak ingin langsung untuk sertai lagi, 5=Sangat ingin untuk sertai lagi	88.9
Burden	Adakah anda mengalami kesukaran untuk menyertai program X-SIAGA? (1=Tiada kesukaran langsung, 5=Sangat banyak kesukaran)	38.9
Ethicality and Cultural Appropriateness	Adakah program X-SIAGA memberi kesan buruk kepada masyarakat Orang Asli? (1=Tidak ada kesan buruk, 5=Ada kesan buruk yang besar)	0.0
	Adakah program X-SIAGA bersesuaian dengan budaya masyarakat Orang Asli? (1=Sangat tidak sesuai, 5=Sangat sesuai)	94.4
Perceived effectiveness	Adakah program X-SIAGA berkesan dalam membantu isi rumah anda bersiap sedia menghadapi Penyakit X dan wabak? (1=Tidak berkesan langsung, 5=Sangat berkesan	94.4
Self-efficacy	Andakah anda yakin anda mampu menggunakan pengetahuan dan kemahiran yang dipelajari daripada program X-SIAGA? (1= Tidak yakin langsung, 5=Sangat yakin)	94.4
Opportunity costs	Adakah program X-SIAGA mengganggu kerja dan keutamaan anda yang lain? (1= Tidak mengganggu langsung, 5=Sangat mengganggu	16.7
General acceptability	Secara am, bagaimanakah penerimaan anda terhadap program X-SIAGA? (1= Sangat tidak baik, 5=Sangat baik)	83.3

Note: *Represents the percentage of responses with ratings of 4 and 5 on the Likert scale.

S4.3 Table. Paired t-test results comparing preparedness scores before and after the X-SIAGA intervention (N=18).

**Outcome**	**Pre**	**Post**	**Mean difference (SD)**	**t Stat**	**95% CI**	**p-value***	**Cohen's d (effect size)**
	**Mean (SD)**	**Mean (SD)**					
Household Preparedness (primary)	0.50 (0.13)	0.72 (0.08)	0.22 (0.11)	-8.44	0.17-0.27	<0.001	1.99
Cognitive Preparedness (secondary)	0.57 (0.12)	0.73 (0.12)	0.16 (0.13)	-5.49	0.10-0.22	<0.001	1.29
Preparedness Behaviour (secondary)	0.43 (0.19)	0.71 (0.08)	0.28 (0.16)	-7.21	0.20-0.35	<0.001	1.70

Note: SD, standard deviation; CI, confidence interval; *level of significance at 0.05.

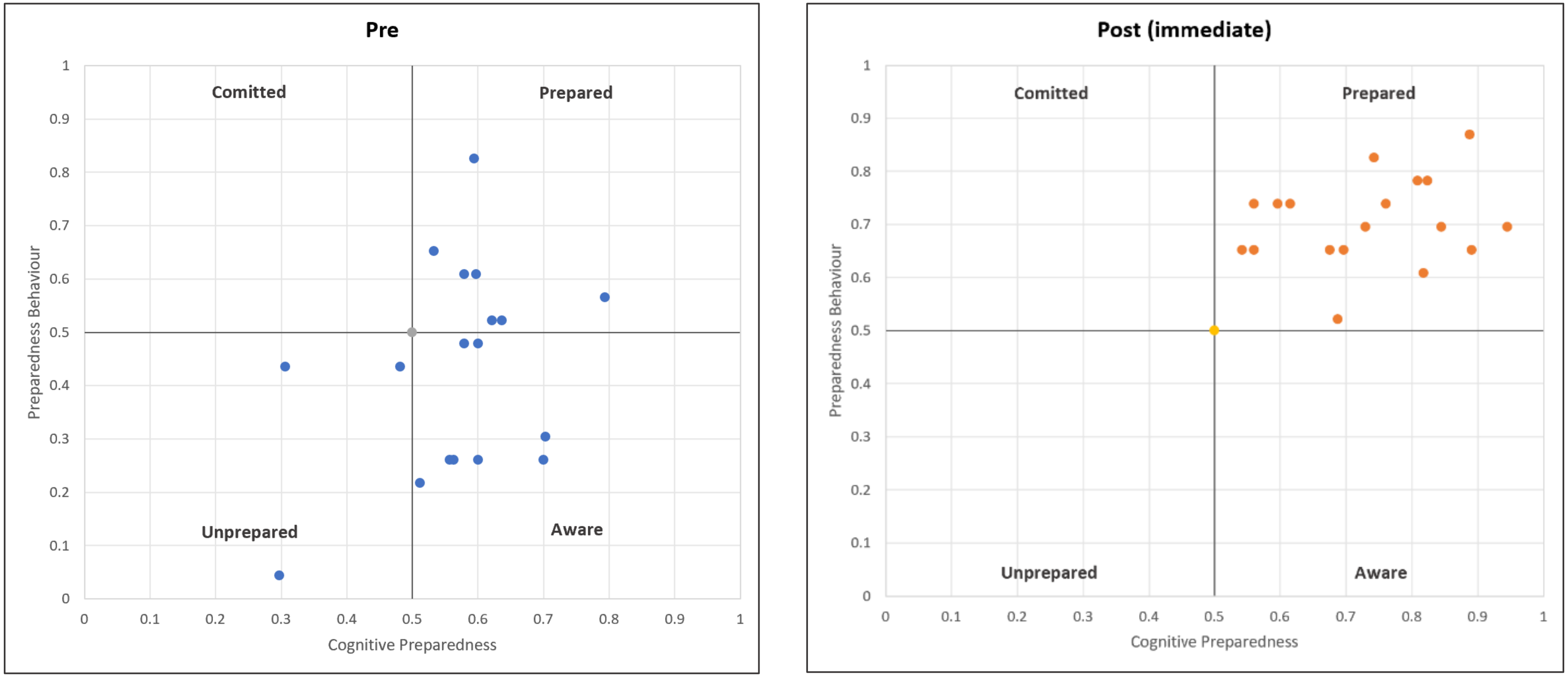

S4 Fig. Matrices comparing household outbreak preparedness scores before and after the X-SIAGA intervention (N=18).
