# Supplementary material for: Development of X-SIAGA: A disease X and outbreak preparedness intervention for indigenous households in Selangor, Malaysia
Source: PLoS One. 2026 Mar 30;21(3):e0345785. doi: 10.1371/journal.pone.0345785 (PMC13035154; doi:10.1371/journal.pone.0345785)
Supplement: Attachment — Submitted filename: My comments_AY.pdf [file pone.0345785.s005.pdf]

The manuscript titled Development of X-SIAGA to improve household preparedness for Disease X and outbreaks among Orang Asli communities in Selangor, Malaysia: Application of the Intervention Mapping framework. The authors provided some nice data about how X-SIAGA is an effective novel intervention strategy to inform household preparedness for disease X outbreaks among the Orang Asli communities in Selangor. Overall, the authors did a nice job in developing comprehensive evidenced based X-SIAGA to map out disease X in the communities. I have some comments and suggestions for the authors to consider in no particular orders.

1. The title is too long, consider shortening the title and retain focus.

2. In line 26-29, consider explaining intervention mapping (IM) approach and give the full meaning of HOPE, since this is their first mention.

3. There are many long sentences in lines 42–55, 67–80, 197–205 which could be summarized for clarity.

4. In Line 344-348, the authors acknowledge planned larger trial but sample size of 18 households is too small. If there is a limitation for the sample size to be limited to 18 households, the limitation should be explained here. A simple statement about the selected sample size, for instance "18 households were considered/selected for the pilot testing out of the 25 households from two Orang Asli Villahes" will provide clarity about the sample size.
5. The authors made concluding statements about X-SIAGA implementation which at this stage of research is not evidence-backed up due to the number of trials. For instance: in line 413-419, the authors wrote "X-SIAGA is prepared for large scale implementation….." this statement could be rephrased to "X-SIAGA shows promise for large-scale implementation"
6. I will suggest that the authors try as much as possible to reduce "the website link references". The authors should look for good references from peer-reviewed publications.
