# Supplementary material for: Development of X-SIAGA: A disease X and outbreak preparedness intervention for indigenous households in Selangor, Malaysia
Source: PLoS One. 2026 Mar 30;21(3):e0345785. doi: 10.1371/journal.pone.0345785 (PMC13035154; doi:10.1371/journal.pone.0345785)
Supplement: Attachment — Submitted filename: Response to Reviewers.pdf [file pone.0345785.s006.pdf]

Thank you, Reviewers, for taking the time to review our manuscript. We have carefully considered each comment and made the necessary changes where we think they are appropriate. Please see the file “Revised Manuscript with Track Changes” to view the changes made, as the line numbers may differ from the manuscript file without track changes. Below are our responses to each of the reviewer comments.

Authors’ response to Reviewer #1 comments:

No.	Reviewer #1 comment	Authors’ response
1.	Introduction<br>The authors effectively sets the stage by defining key concepts (outbreaks,Disease X,preparedness) and estsblishes the vulnerability of the Orang Asli population. However the link the between general outbreak preparedness and the specific focus on "Disease X" looks sightly underdeveloped and also the author should consider briefly mentioning the theoretical frameworks(SCT,HBM) that underpin the study so it prime the readers for the approach used.	We thank the reviewer for this comment. The link between general outbreak preparedness and Disease X has been strengthened (lines 72–82). We did not introduce the theoretical frameworks (SCT, HBM) in the Introduction because the selection and application of these theories are part of Step 2 and Step 3 of the Intervention Mapping framework and are discussed in detail in the Methods and Results sections.
2.	Methods<br>The use of the Intervention Mapping framework is a major strenght and it is well-structured. The matrices in Tables 1& 2 should be looked at again , specify the selection criteria for the expert panel and the community members and clarify how the 18 households were selected.	We appreciate the reviewer’s positive comment on the use of the Intervention Mapping framework. Regarding Tables 1 and 2, we believe the tables are already comprehensive. We would be grateful for more specific guidance if additional changes are needed. As suggested, we have now specified the selection criteria for the expert panel (lines 251–253). We have also clarified how the 18 households were selected in the Methods section (lines 280–283).
3.	Results<br>The validity indexes (S-CVI, S-FVI) are exceptionally high and strongly support the content and face validity of both the intervention and the measurement tool.However the term "significant improvement"<br>is accurate statistically but requires context. What was the baseline score? What is the range the HOPE scale? A mean difference of 0.22 is dificult to interpret without this.If you pre- and post-interventation mean score.	We thank the reviewer for this suggestion. We have added the pre- and post-intervention scores (lines 384–385) and have also included the minimum and maximum HOPE scores in S4 File to provide clearer context for interpretation.

Authors' response to Reviewer #2 comments:

No.	Reviewer #2 comment	Authors' response
1.	The title is too long, consider shortening the title and retain focus.	We agree with this suggestion and have shortened the title accordingly.
2.	In line 26-29, consider explaining intervention mapping (IM) approach and give the full meaning of HOPE, since this is their first mention.	We have added a brief explanation of the IM approach (lines 27-28) and provided the full name of HOPE (lines 31-32) at first mention, as suggested.
3.	There are many long sentences in lines 42–55, 67–80, 197–205 which could be summarized for clarity.	We appreciate this suggestion. We have revised lines 42–55 (now 56–59) and 67–80 (now 97–99). However, for lines 197–205 (now 221–232) this section describes the five core components of X-SIAGA. As the manuscript focuses on the development of the intervention, we consider this level of detail important, and the content is already presented as a concise numbered list. We are concerned that further summarization would dilute important context and reduce readers' understanding. We therefore hope the reviewer will understand our decision to retain this section as it is.
4.	In Line 344-348, the authors acknowledge planned larger trial but sample size of 18 households is too small. If there is a limitation for the sample size to be limited to 18<br>households, the limitation should be explained here. A simple statement about the selected<br>sample size, for instance "18 households were considered/selected for the pilot testing out<br>of the 25 households from two Orang Asli Villahes" will provide clarity about the sample<br>size.	We thank the reviewer for this comment. We have added an explanation of the pilot sample size in the Methods section (lines 280–283).
5.	The authors made concluding statements about X-SIAGA implementation which at this<br>stage of research is not evidence-backed up due to the number of trials. For instance: in	We appreciate this feedback and agree that the wording should be more cautious. We have revised the statements in the abstract (line 42) and conclusion (line 454-455) as suggested.

	line 413-419, the authors wrote “X-SIAGA is prepared for large scale implementation…..”<br>this statement could be rephrased to “X-SIAGA shows promise for large-scale implementation”	
6.	I will suggest that the authors try as much as possible to reduce “the website link references”. The authors should look for good references from peer-reviewed publications.	We thank the reviewer for this observation. Out of 55 citations, only 7 are website references. References [1, 2] are WHO terminologies and statements on outbreaks and Disease X, [6] is FEMA’s definition of preparedness, and [15, 18, 19, 23] are database sources. We consider these appropriate given their authoritative and technical nature, but we will remain attentive to opportunities to replace web sources with peer-reviewed literature where possible in future work.
