# Supplementary material for: Development of X-SIAGA: A disease X and outbreak preparedness intervention for indigenous households in Selangor, Malaysia
Source: PLoS One. 2026 Mar 30;21(3):e0345785. doi: 10.1371/journal.pone.0345785 (PMC13035154; doi:10.1371/journal.pone.0345785)
Supplement: Attachment — Submitted filename: Response to Reviewers_V3.pdf [file pone.0345785.s007.pdf]

Thank you, Reviewers, for taking the time to review our manuscript titled "Development of X-SIAGA: A Disease X and Outbreak Preparedness Intervention for Indigenous Households in Selangor, Malaysia". We have carefully considered each comment and made the necessary changes where we think they are appropriate. Please see the file "Revised Manuscript with Track Changes" to view the changes made, as the line numbers may differ from the manuscript file without track changes. Below are our responses to each of the reviewer comments.

Authors' response to Reviewer #3 comments:

No.	Reviewer #1 comment	Authors' response
1.	Align claims with design: Reframe "effective/ready for implementation" to feasible/acceptable with preliminary improvement, and state it is ready for full-scale trial evaluation, not implementation.	Thank you for your comment. The manuscript has been revised as suggested (lines 41-43, 414 and 473).
2.	Report HOPE instrument details: Clearly describe domains/subscales, item response options, scoring, scale range, handling of missing data, and what the reported mean difference (0.22) represents. Add reliability (e.g., Cronbach's alpha/omega) from the pilot sample.	Thank you for your comment. We have revised the manuscript to more clearly describe the HOPE instrument's domains, item response options, handling of missing data (lines 242-248), and the meaning of the reported mean difference (0.22) (lines 395-396).<br><br>As is now clarified in the Methods section, additional psychometric properties, as well as detailed scoring methods and classification procedures, were not recalculated or described in depth for this small pilot sample, as they have already been comprehensively assessed and reported in a separate validation study using a larger sample (currently under review).
3.	Strengthen statistical reporting: Specify the pre–post test used, assumptions (if relevant), report baseline and post values, 95% CIs, and effect sizes for total and subscales.	Thank you for your suggestion. We have revised the manuscript to specify the use of paired t-tests for pre–post comparisons (lines 277-278). Baseline and post-intervention values, 95% confidence intervals, and effect sizes for household preparedness scores are reported in the Results (lines 36 and 394-395), with these statistics for all outcomes provided in Supplementary File S4.

4.	Improve validity methods transparency: Provide clearer reporting of content/face validity procedures and item/material-level summaries (or accessible supplement), and justify thresholds (especially with small rater numbers).	Thank you for your feedback. We have revised the Methods section to include the thresholds for the validity indices (Lines 269-270). Item- and material-level results for both content and face validity has been provided in Supplementary File S3.
5.	Fix data availability compliance: Replace/augment “available on request” with a clearer sharing pathway, ideally de-identified/aggregated data + codebook in an appropriate repository if ethics permits.	Thank you for your comment. The data generated by this study have been provided in the supplementary files and have also been deposited in the Mendeley Data repository, with the access link provided to the editor.
6.	Reproducibility of intervention: Provide facilitator guidance (manual/scripts), training, and fidelity monitoring tools/checklists; clarify language/reading-level adaptations.	Thank you for your comment. The development process, core components, and material contents has been described in detail throughout the manuscript and in the accompanying tables and supplementary files. We believe this information is sufficient to support adaptation. However, the finalized guidebook and intervention materials are copyrighted. These materials will be made available for purchase through the authors’ institution’s official website.
7.	Clarify participant selection and procedures: Recruitment, inclusion/exclusion, incentives, administration mode (self vs interviewer), and exact timing between pre and post.	Thank you for your comment. Details regarding participant recruitment criteria (lines 250-252, 256-258 and 283-287), administration mode (self-administered; lines 261-262), and the exact timing of pre- and immediate post-assessments (lines 277, 388, 390) has been described in the Methods section. Information regarding incentives has also been added as suggested (lines 287-289).
8.	Expand limitations: Note pre–post threats (testing/Hawthorne), lack of follow-up durability, clustering/context effects, and that changes may reflect short-term knowledge rather than sustained behavior change.	Thank you for your comment. The manuscript has been revised as suggested (lines 453-458).
9.	Minor polish: Ensure consistent terminology and briefly justify potentially non-obvious components	Thank you for your comment. The manuscript has been revised as suggested (lines 374-375).

	(e.g., CPR) and address any acceptability concerns.	
