# Supplementary material for: Development of X-SIAGA: A disease X and outbreak preparedness intervention for indigenous households in Selangor, Malaysia
Source: PLoS One. 2026 Mar 30;21(3):e0345785. doi: 10.1371/journal.pone.0345785 (PMC13035154; doi:10.1371/journal.pone.0345785)
Supplement: Attachment — Submitted filename: Response to Reviewer 5.pdf [file pone.0345785.s008.pdf]

Thank you to the reviewers for taking the time to review our manuscript, titled "Development of X-SIAGA: A Disease X and Outbreak Preparedness Intervention for Indigenous Households in Selangor, Malaysia." We appreciate the reviewers' thoughtful suggestions. However, after careful consideration and discussion, we found that the proposed revisions did not align with the primary objectives of the manuscript, and therefore no changes were made. Below are our detailed responses to the reviewer comments.

Authors' response to Reviewer #5 comments:

No.	Reviewer #5 comment	Authors' response
1.	In line 1<br>Development of X-SIAGA: A Disease X and Outbreak Preparedness Intervention for Indigenous Households in Selangor, Malaysia<br>The title could be more engaging by incorporating action-oriented language or mentioning the anticipated outcomes of the intervention, such as "enhanced health resilience" or "community empowerment.<br>Suggested title:<br>X-SIAGA: Enhancing Household Preparedness for Outbreaks of Disease X Among the Orang Asli in Selangor	Thank you for your suggestions regarding the manuscript title. After careful consideration and discussion with the research team, we have decided to retain the original title, "Development of X-SIAGA: A Disease X and Outbreak Preparedness Intervention for Indigenous Households in Selangor, Malaysia," as we believe it most accurately reflects the scope and developmental focus of this manuscript.<br><br>We appreciate your recommendation to use a more action-oriented title and to mention anticipated outcomes such as enhanced resilience or empowerment. However, since this manuscript focuses on the development process rather than intervention outcomes or effectiveness, we feel it is more appropriate to retain a title that clearly reflects its developmental scope.<br><br>Similarly, we chose to use "Disease X and Outbreak Preparedness" rather than "Outbreaks of Disease X" to indicate that the intervention is intended to prepare communities for a range of possible future threats, including novel pathogens represented by Disease X. We appreciate your input and will certainly take your suggestions into consideration for future manuscripts reporting the results of the effectiveness trial.
2.	We sincerely thank the author for their valuable contribution to the study, which sheds light on critical health challenges faced by vulnerable communities. This	Thank you for your feedback.

	research is vital as it not only addresses the immediate need for outbreak preparedness among the Orang Asli but also serves as a model for similar interventions in other marginalized communities, promoting health equity and resilience.	
3.	The high content and face validity of both X-SIAGA and the HOPE questionnaire indicate strong alignment with community needs and expectations. This validity enhances confidence in the intervention's design and its potential for success in improving preparedness.	Thank you for your feedback.
4.	Continuous evaluation fosters a culture of learning and adaptation, ensuring that the intervention remains relevant and effective. This commitment to evaluation demonstrates responsiveness to community feedback, enhancing trust and collaboration.	Thank you for your feedback.
5.	In line 130<br>• It is notable that the team consists of two public health academics, one public health physician, and one medical officer, along with a member of Orang Asli descent for cultural relevance. However, the absence of a community health or public health nurse is concerning, as their expertise is crucial for addressing practical implementation strategies and enhancing community engagement. Including a nurse could provide valuable insights into frontline health challenges and strengthen the team's overall effectiveness in promoting health within the community.	Thank you for your observation regarding the composition of the Intervention Development Committee (IDC). The IDC was responsible for spearheading and implementing steps 1 to 5 of the intervention mapping process.<br><br>As described in the manuscript, the expert panel for content validation and the Orang Asli community members who participated in face validation were distinct from the IDC. These groups were engaged during program evaluation, which corresponds to step 6 of intervention mapping. The sociodemographic characteristics of the content validation experts are provided in Supplementary File 3 in Table S3.1, while those of the Orang Asli community members involved in face validation are presented in Supplementary Table S3.6.<br><br>Regarding the concern about the absence of a nurse in the development process, we wish to clarify that a nurse was involved in step 6. The face validation group included a nurse (rater #12) and an Assistant Medical Officer (rater #11), whose job scope in the public health field

		in Malaysia closely aligns with that of a nurse. Both individuals are of Orang Asli descent. Their individual ratings are detailed in Supplementary Tables S3.7 to S3.11, and their perspectives were incorporated into the evaluation and refinement of the intervention materials.<br><br>We hope this clarifies the process and the distinct roles of contributors involved in the development and validation of the intervention.
